# Reconstitution of the LnsAB lipoprotein *N*-acylation system complex from *Staphylococcus aureus* and characterization of substrate preference

**DOI:** 10.1016/j.jbc.2026.113434

**Published:** 2026-08-11

**Authors:** John H. Gardiner, Gloria Komazin, Timothy C. Meredith

**Affiliations:** 1 The Huck Institutes of the Life Sciences, The Pennsylvania State University, University Park, Pennsylvania, USA; 2 Department of Biochemistry and Molecular Biology, The Pennsylvania State University, University Park, Pennsylvania, USA

**Keywords:** bacterial lipoproteins, acyltransferase, signal peptide, *Staphylococcus aureus* (*S. aureus*), toll-like receptor 2, acylation, extracellular acylation, CPBP, NlpC/P60, LRAT

## Abstract

In *Staphylococcus aureus*, over 50 different types of lipoproteins are tethered to the membrane surface through acylation of an *N*-terminal cysteine residue where they perform multiple cellular roles, from nutrient transport to displaying virulence factors. Lipoproteins are also key focal points for detection by the toll-like receptor 2 family of innate immunity. While all staphylococci initiate lipoprotein biosynthesis by attaching a thioether-linked diacyl glycerol moiety, subsequent α-amino tailoring occurs in a species dependent manner that attenuates toll-like receptor 2 signaling. A two-gene system encoding an NlpC/P60 superfamily enzyme (LnsA) and a CAAX protease/bacteriocin-processing family integral membrane protein (LnsB) is required for lipoprotein *N*-acylation in *S. aureus*, but little is known regarding the mechanism. Herein, we show that the LnsA *N*-terminal α-helix is a canonical cleaved signal peptide that is unnecessary for membrane retention. We use protein complex modeling with diacylated lipopeptide substrate and substituted cysteine crosslinking to demonstrate a key loop on LnsA interacts with LnsB in a multimeric complex. Using targeted site mutagenesis, a Cys-His catalytic dyad common to NlpC/P60 superfamily members is defined while no CAAX protease/bacteriocin-processing motif residues were essential. Reconstitution using recombinant LnsA and LnsB with lipopeptide substrate confirmed that both proteins are required for catalysis *in vitro*, and that the *SN*_1_ acyl chain of phosphatidylglycerol is the preferred acyl chain substrate donor. This work begins to define the LnsAB complex, and further underscores the rich source of unique acylation biochemistry that has evolved in bacterial lipoprotein *N*-terminal modification pathways.

Bacterial lipoproteins are a large class of lipid-anchored proteins broadly conserved across both Gram-positive and -negative bacteria (1, 2). All lipoproteins have an invariant *N*-terminal cysteine modified with an acylated glyceryl unit obtained from phospholipids and connected in a signature thioether bond. Although all bacteria depend on lipoproteins to perform the numerous cellular functions that occur at the membrane-aqueous environment interface, unique acylation patterns result in genera, species, and even strain-specific chemotypes (3, 4). Structural diversity in large part originates from α-amino tailoring enzymes acting on the *N*-terminal cysteine residue of substrates after membrane export and cleavage of the type II signal peptide (5, 6). In Gram-positive low-GC Firmicutes alone, there are at least three distinct lipoprotein chemotypes (2, 7). In *Bacillus subtilis*, an acetylated heptaprenyl carrier shuttles acetyl units to the extracellular face of the membrane, whereby the integral membrane protein lipoprotein heptaprenyl-acetyl transferase acetylates the α-amino terminus of di-acylated lipoproteins (DA-LPs) to make acetylated lipoproteins (Ac-LP) (8). In *B. anthracis* and *Enterococcus faecalis*, an integral membrane protein lipoprotein intramolecular transacylase moves the *SN*_2_ acyl chain from the thioether-attached acylated glycerol moiety to form an amide and complete the lyso-form (9, 10, 11). In human commensals and opportunistic pathogens such as *Staphylococcus aureus* and *Staphylococcus epidermidis*, triacylated lipoprotein (TA-LP) synthesis requires two distinct genes encoded in separate regions of the chromosome named *lnsAB* (lipoprotein *N*-acylation system) (3). Noncommensal staphylococci, such as *Staphylococcus carnosus*, elaborate Ac-LP (4). Specific lipoprotein tailoring among *Bacillus* sp. and *Staphylococcus* sp. appears to be driven by interactions with the toll like receptor 2 (TLR2) family (4, 8, 12), which are important innate immunity receptors that differentially recognize bacterial lipoprotein chemotypes to trigger host defenses (13, 14, 15, 16). Lyso-form lipoproteins are 100 to 1000 fold less potent TLR2 family receptor ligands than DA-LP counterparts (8, 17). Conversely, TLR2-TLR6 receptor bound Ac-LP boosts the immune response while TA-LP recognized by TLR2-TLR1 heterodimers attenuates innate and adaptive immune responses in staphylococci (4).

The mechanism of lipoprotein *N*-acylation in staphylococci is poorly understood (3). LnsAB mediated acylation is entirely unique in seeming to require two separate gene products. LnsA is only found in TA-LP forming genomes of select staphylococci species in a shared genome context (3). At 189 amino acids long, LnsA belongs to the NlpC/P60 superfamily, a diverse group of extracellular hydrolases, peptidases, amidases, and acyl transferases (18). LnsA shares distant sequence similarity to the well-studied microsomal enzyme lecithin:retinol acyltransferase (LRAT), which catalyzes acyl transfer from the *SN*_1_ position of phosphatidylcholine to all-*trans*-retinol acceptor in the retinal pigmented epithelium of the eye (19). LnsB is a member of the CAAX protease and bacteriocin-processing enzyme (CPBP) family (20), a large class of polytopic integral membrane proteins encompassing metalloproteases and other proteins of poorly defined cellular function. CPBP family enzymes are in all staphylococci species, with multiple paralogues often present in a given genome (at least six in *S. aureus*). Curiously, CPBP family ORFs syntenic with *S. aureus lnsB* are present in other staphylococci that do not make TA-LP, such as Ac-LP synthesizing *S. carnosus* (3). Once more, LnsA and LnsB share no appreciable sequence similarity to either of the well-studied lipoprotein *N*-acyl transferase enzyme Lnt from *Escherichia coli* (21, 22, 23) or the TA-LP forming enzyme Lnb from *Bacteroides* (24).

Herein, we report the characterization of the LnsAB lipoprotein *N*-acylation system from *S. aureus*. We show LnsA is exported and remains associated with the membrane after cleavage of the signal export peptide. We use substituted cysteine crosslinking informed by AlphaFoldv3 (25) and Protenix (26) with DA-LP ligand modeling to provide evidence that LnsA physically interacts with LnsB in a multimeric complex on the extracellular membrane surface. Complex formation between LnsA and LnsB is shown to be important for catalysis, as *S. epidermidis* LnsA cannot substitute for the *S. aureus* LnsA ortholog in TA-LP synthesis unless a key stretch of amino acids in loop 4 at the putative complex interface is maintained in a LnsA chimera. Targeted mutagenesis of the putative LnsA active site points to key catalytic residues, including a His/Cys dyad conserved with LRATs. Conversely, it is shown that the signature CPBP motifs in LnsB are nonfunctional with respect to TA-LP synthesis. Finally, we use a reconstituted LnsA/LnsB system with fluorescently labeled DA-LP∗ acceptor substrate to show the *SN*_1_-position of phosphatidylglycerol (PG) is the preferred acyl chain source and hence dictates the TA-LP chemotype heterogeneity detected by TLR2.

## Results

### Distribution of LnsA and LnsB orthologs in staphylococcal species

**Figure 1:**
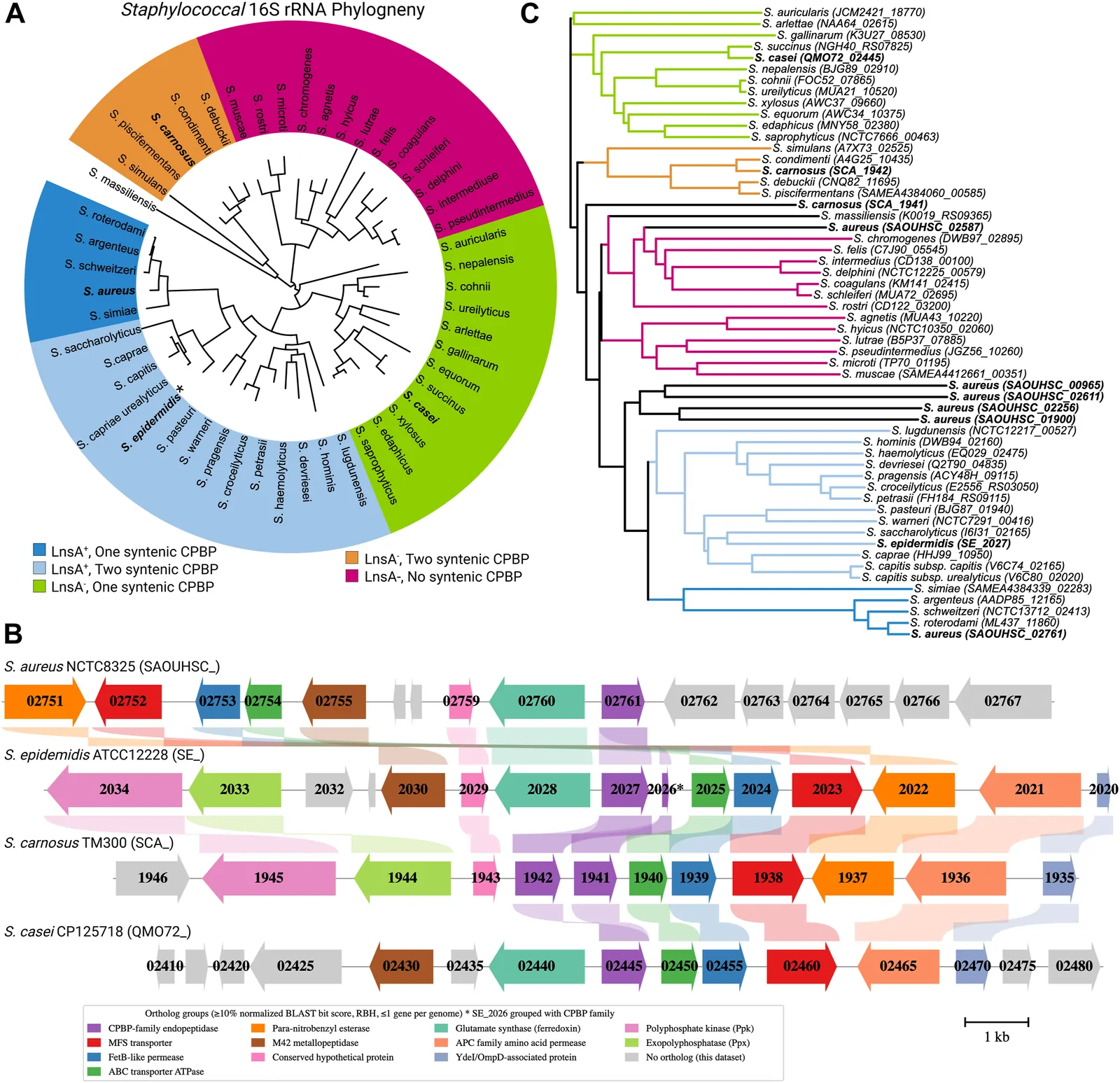
**Phylogenomic distribution of LnsA and LnsB orthologs in staphylococcal species.***A*, the phylogenetic tree of representative staphylococcal species was built from ClustalOmega alignment of 16S rRNA nucleotide sequences. Orthologs of LnsA were identified using query LnsA protein sequences (SAOUHSC_00822 from *S. aureus* and SE_0583 from *S. epidermidis*) against entire genomes (LnsA^+^, *light* and *dark blue* shaded). Syntenic CPBP family ORFs were scored using LnsB loci from *S. aureus* (SAOUHSC_02761) and *S. epidermidis* (SE_2027), and grouped according to LnsA^+^ with either one (*light blue*) or two (*dark blue*) syntenic CPBP ORFs. The LnsA^-^ species were likewise grouped, with one (*green*), two (*orange*), or no (*magenta*) syntenic CPBP ORFs. Bold texted species names were further analyzed for *lnsB* gene synteny in *panel B*. *B*, gene loci from representative species belonging to each LnsA^+/−^ CPBP synteny clade were selected and are centered on *lnsB* from *S. aureus* (SAOUHSC_02761, *purple* shaded). ∗ - SE_2026 CPBP ORF from *S. epidermidis* encodes a pseudo-gene that is truncated due to an internal frameshift. *C*, protein sequences of CPBP proteins (locus tags in parenthesis) from each species used in *panel A* were compared for sequence similarity. All *S. aureus* CPBP orthologs, all those syntenic with LnsB for species represented in *panel B*, and the most similar CPBP orthologs from species lacking LnsB synteny are included for comparison (*bold* text). Clade coloring is according to *panel A*. CPBP, CAAX protease and bacteriocin-processing; Lns, lipoprotein N-acylation system.

We previously proposed that LnsA and LnsB are both necessary for TA-LP biosynthesis (3). To begin to understand why syntenic CPBP orthologs are present and whether species specific lipoprotein chemotypes can be correlated to *lnsAB* genetic determinants across the genus, we overlaid *lnsAB* genotypes according to species relatedness based on 16S rRNA (Fig. 1*A*). Genomes harboring high ranking LnsA^+^ orthologues (>20% amino acid sequence identity with *S. aureus* LnsA) were confined to a subset of closely related species, while CPBPs hits were identified in all genomes (Fig. S1). The LnsA^+^ species further divide into two sub-clades, encoding one or two syntenic CPBP copies. While most of the latter sub-clade retains both CPBPs apparently intact, one copy is a pseudogene in *S. epidermidis*. The LnsA^-^ species also coclustered tightly, with distinct clades harboring two, one, or no syntenic CPBPs (Fig. 1*B*). Pairwise alignment scores of staphylococcal CPBPs with *S. aureus* LnsB, which ranged from 20 to 35% sequence identity once outside of the *S. aureus* clade, were imperfect predicters of synteny and cooccurrence of LnsA^+^ (Fig. S1). Multi sequence alignment (MSA), however, clearly separated LnsB-type CPBPs within LnsA^+^ species from other CPBPs, including CPBP paralogs in *S. aureus* and notably from syntenic copies in other species (Fig. 1*C*). This suggests that while genome synteny and %-sequence identity/similarity alone are not enough to assign LnsB function at the species level, MSA analysis can help define LnsB activity and predict TA-LP biosynthesis.

### Cross-species complementation of TA-LP biosynthesis supports a LnsB CPBP sub-clade

**Figure 2:**
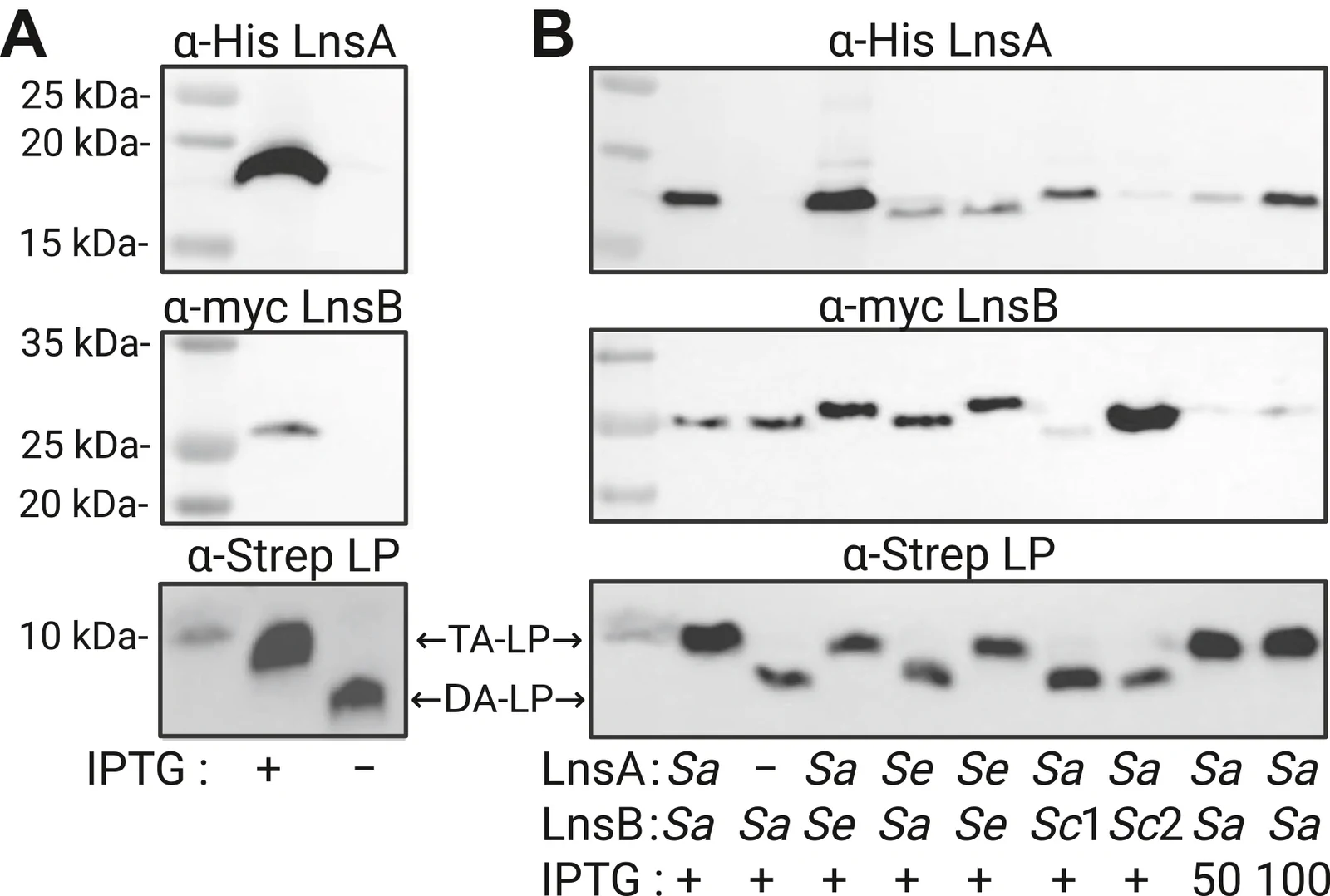
**SDS-PAGE analysis of LP acylation states after cross-species LnsA/LnsB complementation.***A*, a plasmid encoded, IPTG-inducible copy of *S. aureus N*-terminal His tagged LnsA (SAOUHSC_00822) and *C*-terminal α-myc tagged LnsB (SAOUHSC_02761) controls both protein expression (*top* and *middle* panels, respectively) and the synthesis of TA-LP from a *C*-terminal strep tagged DA-LP fragment encoded on a second plasmid in strain GKM2702. *B*, combinations of strains with LnsA from *S. aureus* (*Sa*, SAOUSC_00822) and *S. epidermidis* (*Se*, SE_0583) expressed in tandem with the syntenic LnsB/CPBP orthologs from *S. aureus* (*Sa*, SAOUHSC_02761), *S. epidermidis* (SE_2027), or *S. carnosus* (*Sc1*: SCA_1941 and *Sc2*: SCA_1942) were tested for TA-LP synthesis. From *left* to *right*: GKM2702, TXM2829, TXM2874, TXM2875, TXM2876, TXM2879, and TXM2880. Strains were grown with 1 mM IPTG inducer (+), unless indicated (50 and 100 μM). CPBP, CAAX protease and bacteriocin-processing; DA-LP, di-acylated bacterial lipoprotein; Lns, lipoprotein N-acylation system; TA-LP, tri-acylated bacterial lipoprotein.

To test the function of a subset of staphylococcal CPBPs, we introduced candidate *lnsB* CPBP genes into an *S. aureus* Δ*lnsB* host under control of an IPTG-inducible promoter and assayed for TA-LP synthesis (Fig. 2). Consistent with prior gene deletion studies (3), IPTG was required for conversion of a plasmid encoded strep tagged DA-LP lipopeptide reporter to TA-LP (Fig. 2*A*). The *S. epidermidis* LnsB, which is syntenic with *S. aureus* (Fig. 1*B*), was fully functional and restored TA-LP synthesis when paired with *S. aureus* LnsA (Fig. 2*A*, *bottom panel*). In contrast, *S. epidermidis* LnsA was unable to partner with *S. aureus* LnsB as the lipopeptide remained di-acylated despite the protein being successfully expressed (Fig. 2*B*, *top panel*). *S. epidermidis* LnsA was catalytically competent with *S. epidermidis* LnsB, however, ruling out factors unique to the *S. aureus* cell envelope that may have prevented functional complementation. Expression of either syntenic CPBP from *S. carnosus* did not yield TA-LP. While the failure of LnsB/CPBP orthologues to complement may have arisen from protein instability or low expression, down titration of *S. aureus* LnsAB to comparable protein levels had no effect on TA-LP formation. The results are consistent with the functional delineation of the LnsB sub-clade within the CPBP family by MSA (Fig. 1*C*).

### The LnsA *N* terminus is a cleaved signal peptide that is not required for membrane association

**Figure 3:**
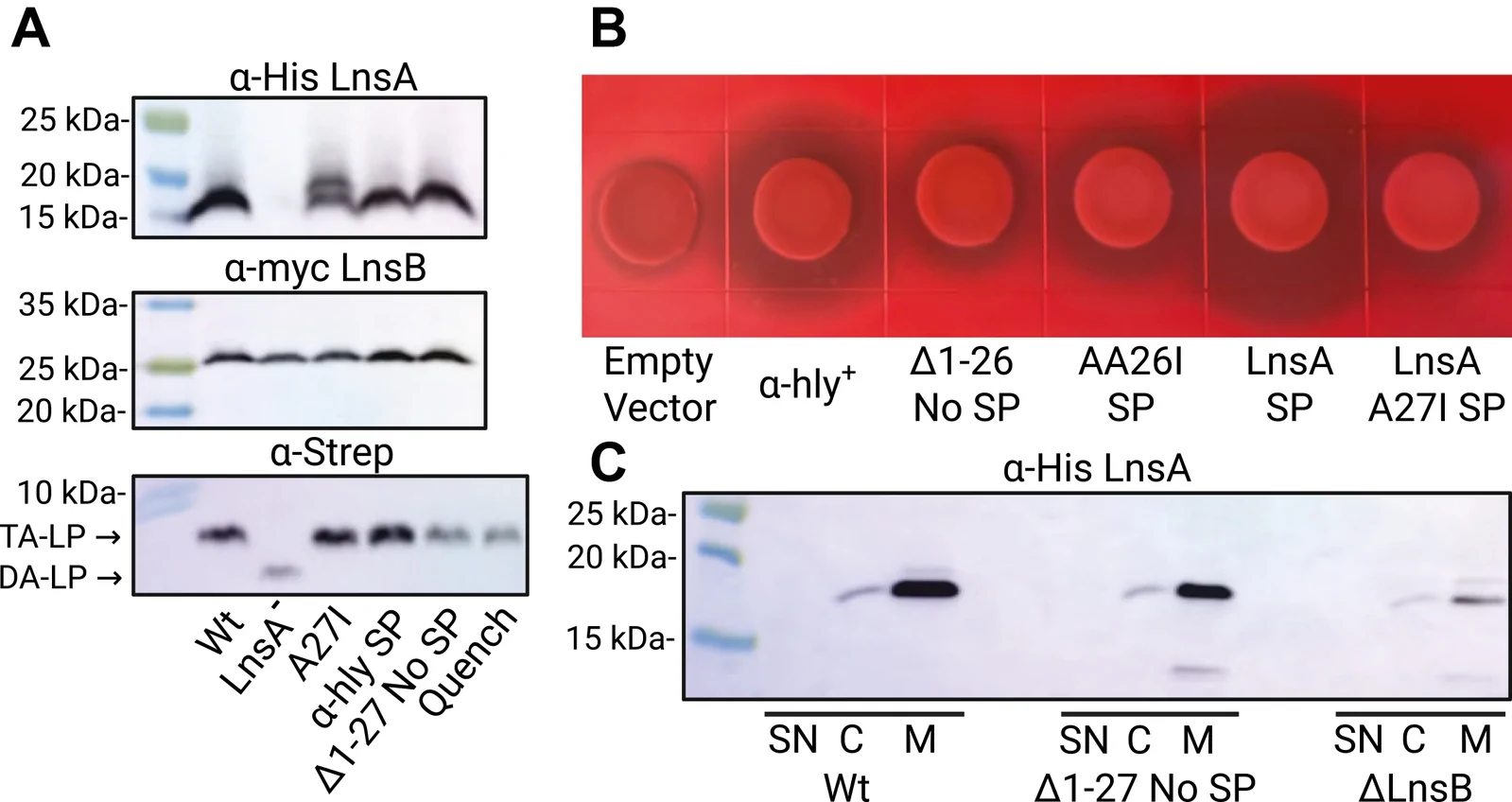
**The *N*-terminal a-helix of LnsA is a cleaved signal peptide.***A*, constructs lacking LnsA (LnsA^−^, TXM2829), with A27I in the −1 position of the putative signal peptidase cleavage site (A27I, TXM2855), with the 26 amino acid long α-hemolysin signal replacing the native LnsA *N* terminus peptide (α-hly SP, TXM2857), or with no encoded signal peptide (Δ1-27 No SP, TXM2859) were analyzed for TA-LP synthesis by comparison to WT(GKM2702). Where indicated, cellular metabolism was first quenched before preparing extracts for SDS-PAGE analysis. *B*, hemolysis zones were measured on TSA agar plates supplemented with 5% rabbit blood. Strains contained an empty plasmid vector (JG2431), α-hemolysin (SAOUHSC_01121) with a native signal peptide (α-hly, TXM2885), no signal peptide (Δ1-26 No SP, TXM2886), a −1/+1 position signal peptidase target site mutation (AA26I SP, TXM2887), with the 27 amino acid long LnsA leader peptide replacing the native signal peptide in a LnsA/α-hemolysin chimera (LnsA SP, TXM2888), or with a mutation in the −1 position of the signal peptidase target in this chimera (LnsA A27I SP). *C*, cell cultures were fractionated into secreted protein supernatants (SN) and cell pellets, which were further processed into cytosolic (C) and membrane (M) associated fractions by ultracentrifugation. Protein fractions were concentrated to the same final volume, with extracts from cells expressing native LnsA (GKM2702), lacking the SP (Δ1-27-TXM2859), or native LnsA in the absence of LnsB (ΔLnsB, TXM2878) separated by SDS-PAGE and probed for LnsA by α-His immunoblotting. Lns, lipoprotein N-acylation system.

The inability of *S. epidermidis* LnsA to work with *S. aureus* LnsB, despite sharing 60% identity/75% similarity with *S. aureus* LnsA (Fig. S2), suggested LnsA and LnsB proteins must interact to be active. If each protein acted independently in a sequential fashion, this would not be the expectation. Indeed, previous AlphaFold modeling studies suggested LnsA and LnsB may form a protein complex (1). Modeling could not discriminate if the *N*-terminal α-helix is a cleaved signal peptide or is retained to help anchor LnsA to the surface and/or help interface with the integral membrane protein partner LnsB. This is an especially pertinent question considering that, like LnsA, LRAT belongs to the NlpC/P60 enzyme family (18) and contains an *N*- and *C*-terminal α-helical transmembrane domain for membrane retention (27). We first tested LnsA with a mutation in the putative signal peptidase cut site at the −1 position predicted by SignalP 6.0 (28). The A27I mutation produced a larger LnsA isoform (Fig. 3*A*), consistent with slowed signal peptide processing due to the bulky isoleucine side chain. Exchange of the first 26 amino acid signal peptide from α-hemolysin (α-hly), a well characterized secreted protein (29), for the native LnsA leader peptide yielded a single band similar in size to both the WT and to a construct with the signal peptide removed altogether (Δ1-27 No SP). We then performed the corollary experiment with α-hly to see if the LnsA leader peptide could support secretion Fig. 3*B*). Removal of the α-hly signal peptide (Δ1-27 No SP) or substitution of bulky hydrophobic residues in the −1 peptidase cut site (AA26I) markedly decreased the hemolysis halo on blood agar plates, while exchange with the LnsA signal peptide restored α-hly secretion and was functional for export. To confirm the signal peptide cleavage site, His tagged LnsA was expressed in *S. aureus*, isolated, and subject to Edman degradation sequencing. Eight cycles yielded H_2_N-DNXYSEIQ, in good agreement with proteolysis of the signal peptide between the 27/28th amino acid (Fig. S2).

Without the *N* terminus, LnsA does not have any predicted transmembrane α-helices yet constructs (including when the signal peptide was not encoded, see below) still produced TA-LP and hence remained membrane-associated. To investigate, we fractionated cells expressing WT LnsA, LnsA lacking signal peptide, and LnsA in a ΔLnsB background into secreted supernatant, cytosolic, and membrane associated fractions (Fig. 3*C*). In all cases, LnsA was not detected in the secreted supernatant with the bulk of LnsA remaining associated with the membrane. Collectively, the data indicate LnsA encodes a canonical signal peptide removed by proteolysis after transport but stays physically associated with the membrane surface independent of a transmembrane domain or LnsB protein interaction.

### The LnsA-LnsB *in silico* heterodimer model suggests LnsA caps LnsB on the extracellular membrane surface

**Figure 4:**
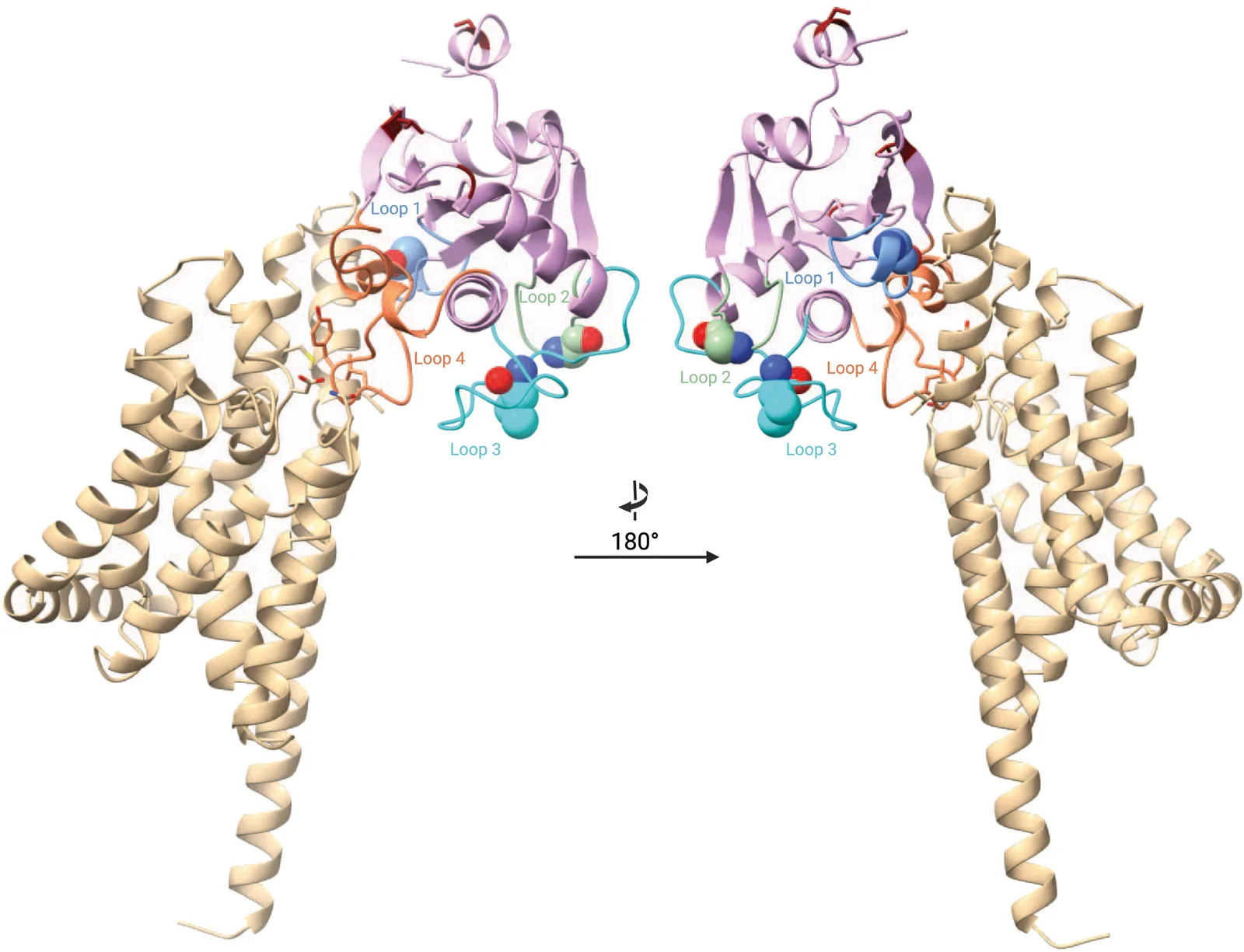
**AlphaFoldv3 model of LnsA-LnsB from *S. aureus*.** Protein sequences for LnsA (lacking signal peptide, *plum*)-LnsB (*tan*) from *S. aureus* were modeled using AlphaFoldv3 (24). The four unstructured LnsA loops are *colored*, with loop-LnsB interface residues targeted for crosslinking indicated (Loop 1, Gly56, atom space filled in *blue* with LnsB Val52, stick in *tan*; see Fig. 7) and Loop 4 (Tyr163/Leu165, stick in *orange* with LnsB Asp139/I40/M67, stick in *tan*; see Fig. 6). Residues on Loop 2 (Gly77, atom space filled in *green*) and Loop 3 (Leu130, atom space filled in *cyan*) are shown for orientation. The three surface exposed LnsA residues (Ser27, Gly109, Val186) targeted for labeling with maleimide-PEG are indicated (*dark red*, see Fig. 5). Lns, lipoprotein N-acylation system.

To begin to probe the nature of the LnsA-LnsB complex, we used AlphaFoldver3 (25) to model various LnsA-LnsB/CPBP permutations (Table S3). Retention or removal of signal peptide had minimal impact on scores or overall trends, consistent with earlier studies (1). Of the six total CPBP partners in *S. aureus*, LnsA-LnsB complexes scored the highest with respect to both pTM (0.84) and ipTM (0.81) (Fig. 4). Alphafoldver3 was unable to discriminate between the active and inactive *S. aureus* and *S. epidermidis* mixed species LnsAB heterodimers, with scores approximating *S. aureus* or *S. epidermidis* LnsAB single species complexes. Protein complex interface scores with either *S. carnosus* syntenic CPBP were low confidence (ipTM = 0.12/0.16); however, consistent with lack of functional complementation (Fig. 2).

**Figure 5:**
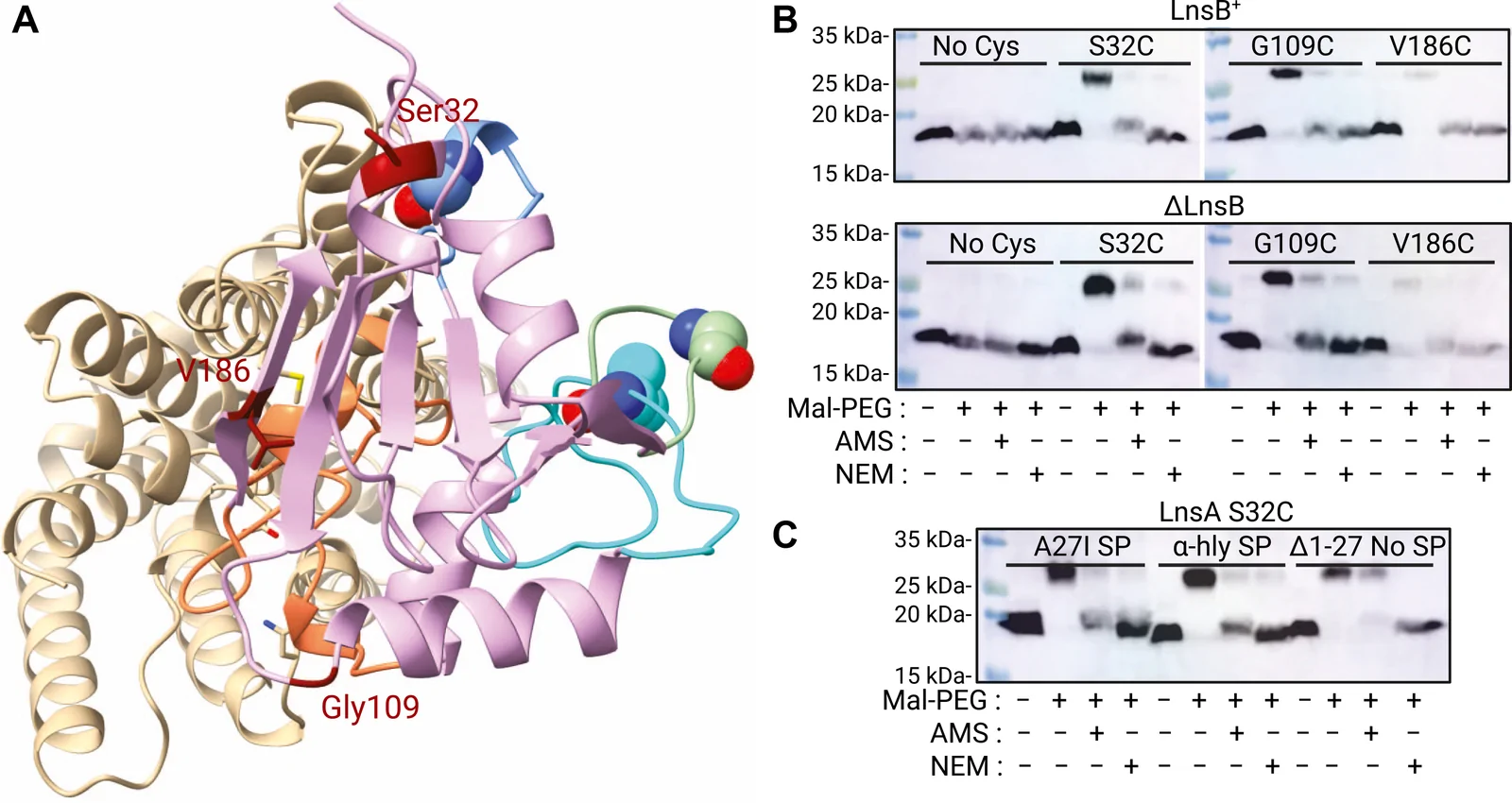
**LnsA export and surface exposure is independent of LnsB and enhanced by the signal peptide.***A*, *top-down* view of the modeled LnsA-LnsB complex (see Fig. 4), with residues mutated to cysteine and targeted for mal-PEG (∼5 kDa) labeling indicated (*dark red*). *B*, SCAM labeling with membrane impermeable (AMS) and permeable (NEM) blocking agents was performed on live cells expressing LnsA variants lacking all cysteine residues except for the substitution site. LnsB was either coexpressed (LnsB^+^ in strains TXM2722, TXM2791, TXM2792, and TXM2800, *top panel*) or not included (ΔLnsB in strain TXM2820, TXM2821, TXM2822, and TXM2823, *bottom panel*). *C*, signal peptide variants in LnsA with a mutated signal peptidase cut site (A27I SP, TXM2856), α-hly SP (α-hly SP, TXM2858), or no signal peptide (Δ1-27 No SP, TXM2860) were labeled with mal-PEG as in *panel B*. α-hly, α-hemolysin; Lns, lipoprotein N-acylation system; mal-PEG, maleimide-PEG.

The AlphaFold LnsA-LnsB model (Fig. 4) was first tested for transport and accessibility of residues on the apical face of LnsA (Fig. 5*A*). The observation that cells could still synthesize TA-LP when expressing LnsA with no signal peptide (Fig. 3*A*, *bottom right panel*) was somewhat surprising given the pose of the putative protein complex. Quenching live cells with organic solvents did not prevent TA-LP formation, ruling out translocation of LnsA lacking signal peptide from the inner face of the cytoplasmic membrane after cell lysis. Three LnsA residues (S32, G109, V186) predicted to be surface exposed were mutated to cysteine to verify extracellular localization by the substituted cysteine accessibility method (SCAM) (30, 31). In all cases, the membrane impermeable blocker 4-acetamido-4′-maleimidylstilbene-2,2′disulfonic acid (AMS) was as effective as the membrane permeable *N*-ethyl maleimide blocker in preventing maleimide-PEG (mal-PEG) modification of the introduced cysteine residues (Fig. 5*B*, *top panel*). Deleting LnsB did not impact surface exposure or accessibility as judged by PEG labeling (Fig. 5*B*, *bottom panel*). We repeated SCAM with the S32C mutation and signal peptide variants to test if LnsA export was somehow independent of signal peptide (Fig. 5*C*). While the A27I signal peptide cut site mutation and α-hly signal peptide exchange remained accessible to the AMS blocker as would be expected if exported, deletion of the signal peptide significantly decreased AMS mediated labeling and protection from mal-PEG. The data indicate the LnsA signal peptide is needed for efficient transport across the membrane, albeit a fraction can evidently transit the membrane without a signal peptide to complete lipoprotein *N*-acylation (Fig. 3*A*, *bottom right panel*).

### Loop 4 of LnsA contacts LnsB at the protein-protein interface in a multimeric complex

**Figure 6:**
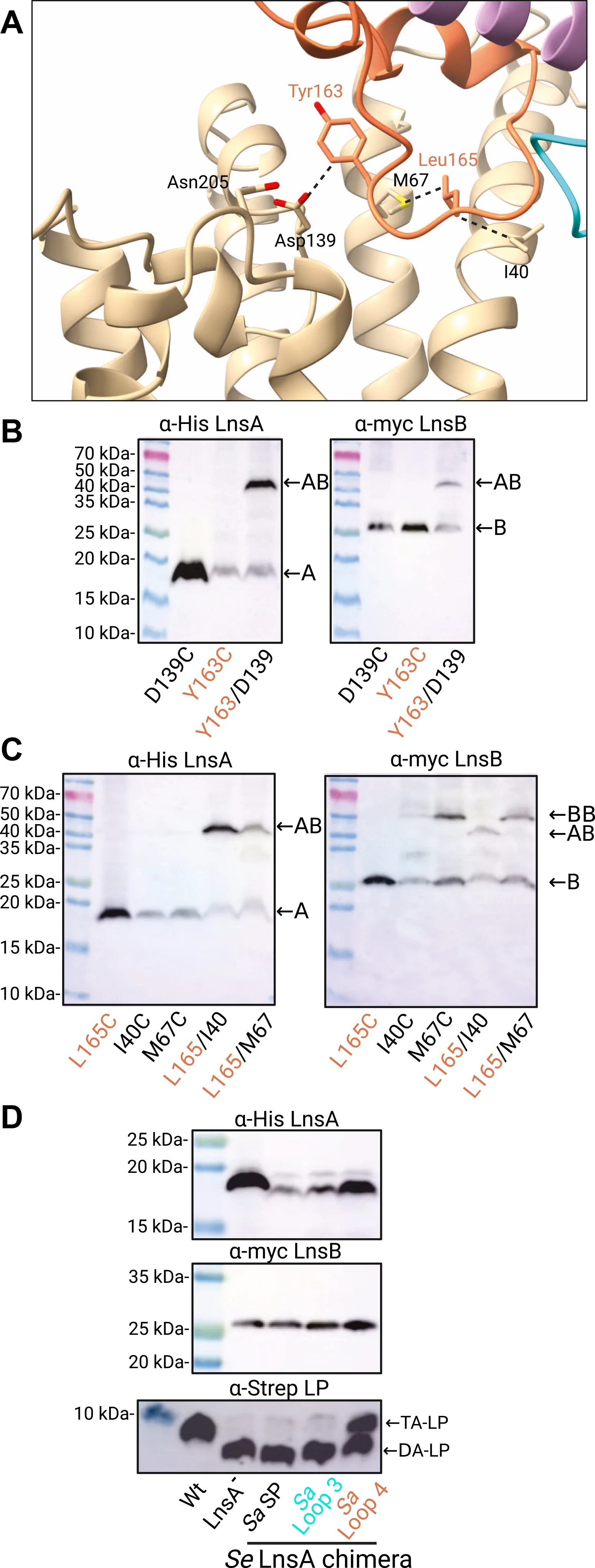
**Cysteine substitution crosslinking and loop 4 exchange between *S. aureus* and *S. epidermidis* indicates a LnsA–LnsB protein interaction.***A*, magnified view of the Alphafoldver3 modeled (see Fig. 4) LnsA (*plum*)-LnsB(*tan*) interface with residues mutated to cysteine for crosslinking shown for loop 4 of LnsA (stick side chain representation, *orange*) and LnsB (stick side chain representation, *tan*). *B*, strains with LnsA and LnsB variants lacking all cysteines except the indicated residues (residues mutated to Cys on LnsA in *orange* text, while LnsB residues are in *black* text) were expressed alone (GKM2816, GKM2818) or in pairs (GKM2817) and oxidized by disulfide exchange using 4-DPS as catalyst. LnsA (α-his), LnsB (α-myc), and resulting protein complexes were separated by nonreducing SDS-PAGE and detected by immunoblotting. *C*, crosslinking of substituted cysteine mutants was performed as in *panel B*, with L165C LnsA cysteine substituted mutants crosslinked to either I40C (*lane 4*, TXM2797) or M67C (*lane 5*, TXM2798) belonging to anti-parallel α-helices in LnsB. Control samples with a single cysteine substitution in LnsA or LnsB are shown (*lanes 1*–3, strains TXM2787, TXM2788, TXM2790). *D*, the lipoprotein acylation activity of *S. aureus* expressing *Sa* LnsA (Wt), lacking LnsA (LnsA^-^), or with *S. epidermidis* (*Se*) LnsA chimeras encoding the *S. aureus* LnsA signal peptide (*Sa* SP, TXM2927), loop 3 (*Sa* Loop 3, 15 amino acids exchanged, TXM2928), or loop 4 (*Sa* Loop 4, 25 amino acid exchanged, TXM2929) substituted were measured after separating chemotypes by SDS-PAGE and immunoblotting for strep tagged TA-LP as in Figure 2. Lns, lipoprotein N-acylation system.

The predicted LnsA-LnsB complex has four main loops, with loop 4 making multiple contacts with LnsB (Fig. 4). We thus first targeted residues within loop 4 for cysteine disulfide crosslinking (Fig. 6*A*). When LnsA Tyr163 or LnsB D139 alone was converted to cysteine, neither protein was mass shifted when 4,4′-dithiopyridine (DPS) was added to catalyze disulfide bond exchange (Fig. 6*B*). A band consistent in size with a LnsA-B heterodimer was observed after DPS treatment when immunoblotting for LnsA or LnsB when both residues were mutated, consistent with their relative proximity predicted in the AlphaFold model. We further tested loop 4 by substituting LnsA L165 with cysteine and sequentially testing for crosslinking to residues located on two additional LnsB α-helices (LnsB I40 and M67). In both cases, heterodimeric LnsA-B complexes were likewise detected by immunoblotting for LnsA but only when both positions were substituted with cysteine (Fig. 6*C*). Collectively, this data suggest that loop 4 does indeed make extensive contacts with LnsB and that the inability of *S. epidermidis* LnsA to interface with *S. aureus* LnsB likely originate from subtle differences in respective loop 4 compositions. We thus constructed *S. epidermidis* LnsA chimeras, exchanging the signal peptide, loop 3, or loop 4 with the corresponding *S. aureus* sequence (Fig. 6*D*). The loop 4 exchange chimera (involving a 25 amino acid stretch encompassing 11 nonidentical amino acids, Fig. S2) partially restored TA-LP synthesis, further supporting a key role for loop 4 in defining the protein-protein interface.

**Figure 7:**
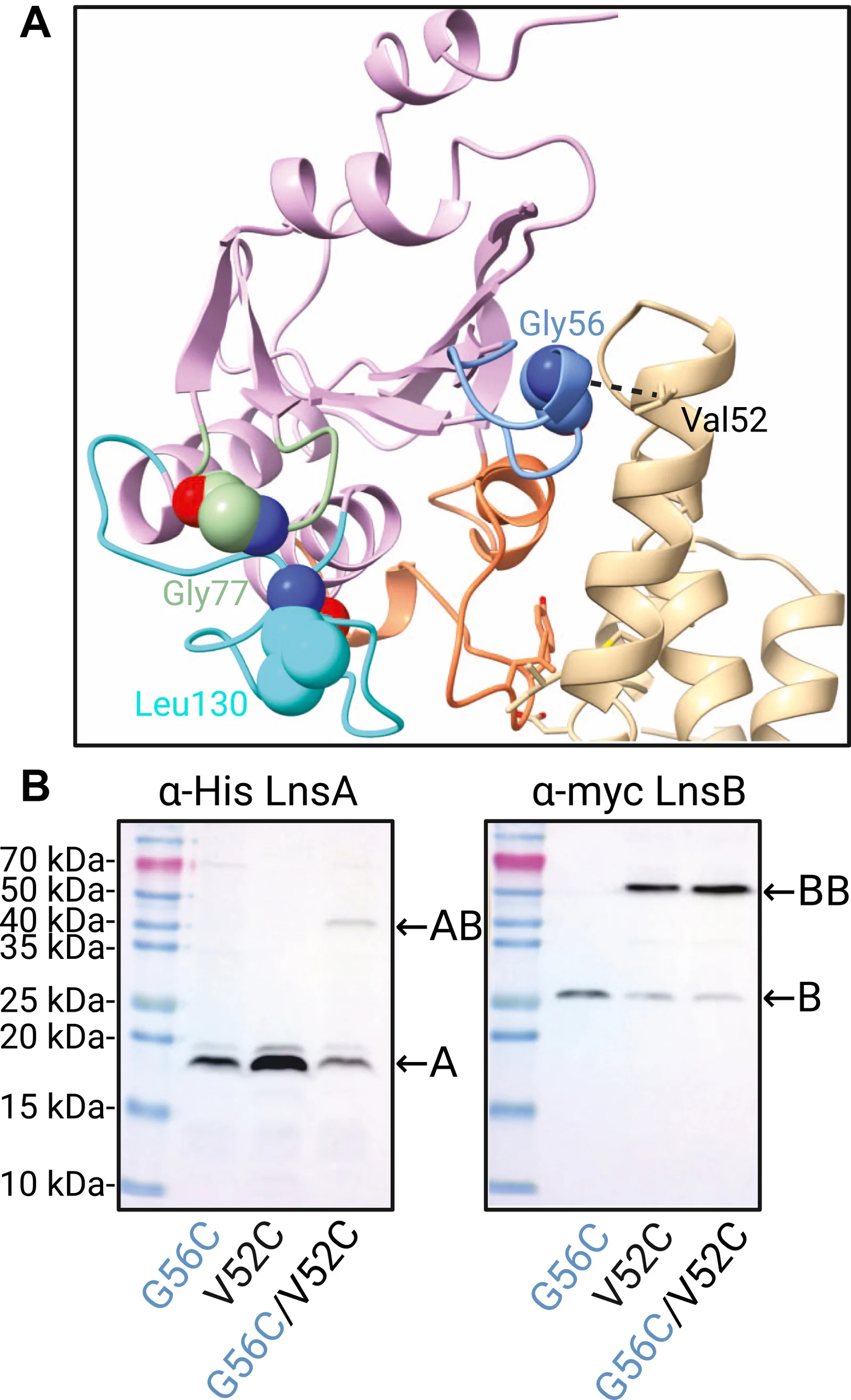
**Homomeric cysteine substitution crosslinking suggests LnsA-LnsB is a multimeric complex.***A*, magnified view of the Alphafoldver3 modeled (see Fig. 4) LnsA (*plum*)-LnsB(*tan*) interface with residues mutated to cysteine for crosslinking shown in loop 1 of LnsA (space filled, Gly56, *blue*) and LnsB (stick side chain representation, Val52, *tan*). *B*, strains expressing LnsA and LnsB variants lacking all cysteines except LnsA G56C (*blue* text, TXM2925), LnsB V52C (*tan* text, TXM2777), or with both substitutions (TXM2727) were crosslinked and probed by immunoblotting as described in Figure 6. Lns, lipoprotein N-acylation system.

Although LnsB M67C did crosslink to LnsA L165C as predicted by their relative proximity in the model, a band higher in molecular weight than the LnsA-B heterodimer was also observed (Fig. 6*C*). The mass shift occurred in the absence of a cognate cysteine on LnsA, indicating the mass shift likely stemmed from LnsB-B homodimeric crosslinks. Potential for a multimeric LnsA-LnsB complex using AlphaFold increased confidence scores, however (Table S3). We turned to a second protein structure prediction method using Protenix (26), which importantly allows inclusion of custom substrates into the model. When a DA-LP (di-palmitoyl *S*-glyceryl-Cys-Ser-Lys-Lys-Lys-Lys) lipopeptide substrate ligand was incorporated in the LnsAB complex model, pTM/ipTM confidence scores consistently improved. Notably, multimeric complexes (LnsA_4_-B_4_ to LnsA_6_-B_6_) with DA-LP ligand outperformed the simple LnsA-B heterodimer, with or without ligand. We chose the LnsA_5_-B_5_ configuration to use as the model for further crosslinking analysis (Fig. S3). In the multimeric model, LnsB V52 on neighboring subunits are in close proximity to each other facing a central cavity and to G56 in loop 1 from LnsA (Fig. 7*A*). Heterodimeric LnsA-LnsB crosslinks were readily observed when probing for His tagged LnsA (Fig. 7*B*). Although we could not confidently assign heteromeric complexes when probing with the α-myc LnsB antibody (likely due to less sensitivity of this antibody system), especially prominent homomeric LnsB-B dimers were detected even if only LnsB V52C was substituted (Fig. 7*B*). While the precise subunit stoichiometries of the complex remain speculative, the cysteine substitution crosslinking results strongly support a multimeric arrangement of LnsA-LnsB heterodimers.

### The LnsA active site shares a catalytic Cys/his dyad while LnsB CPBP motifs are nonessential

**Figure 8:**
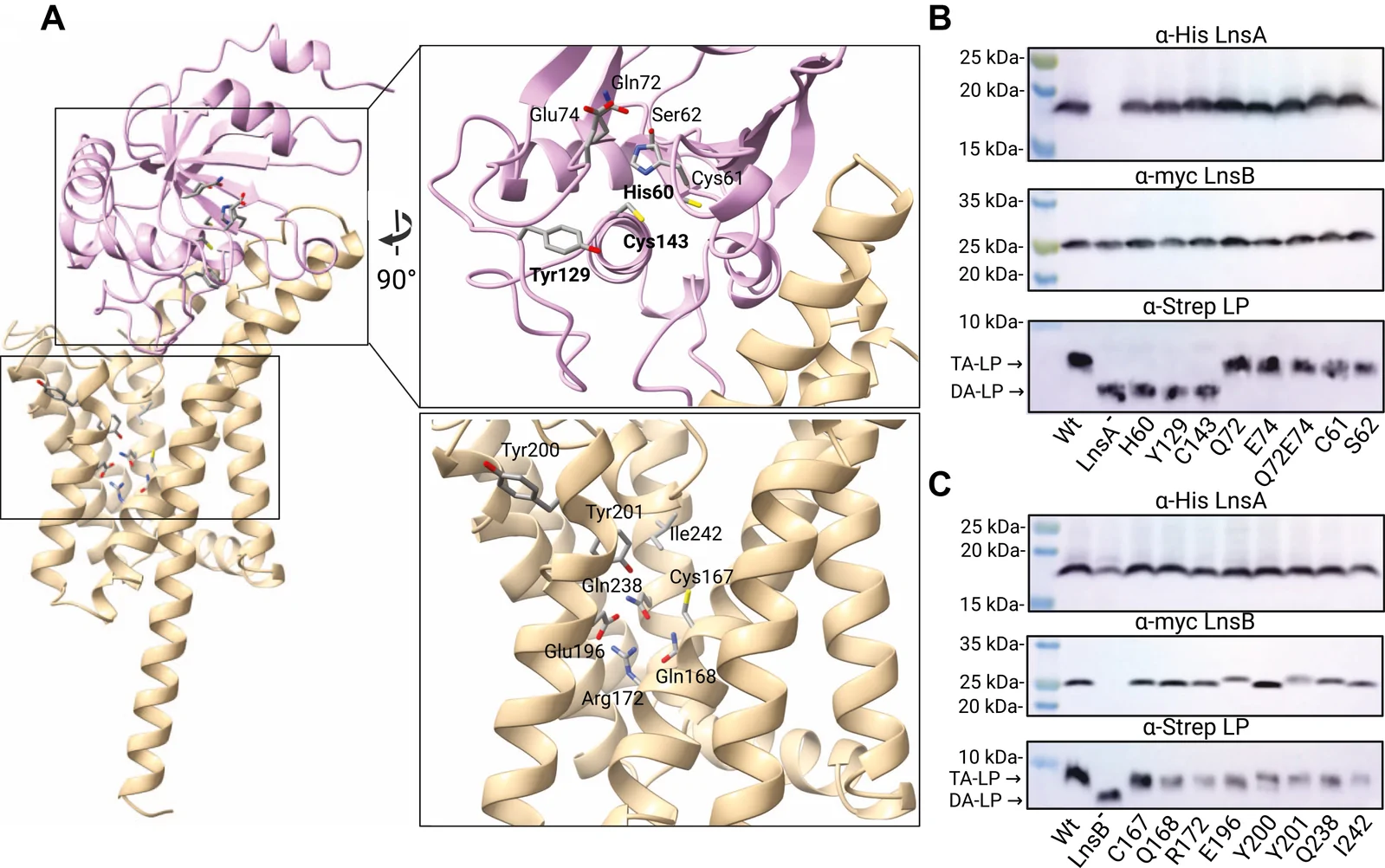
**LnsA shares key active site residues with LRAT family proteins while CPBP family motif residues are nonfunctional in LnsB.***A*, magnified view of the Alphafoldver3 modeled (see Fig. 4) putative active sites of LnsA (*plum*) and LnsB(*tan*), with residues targeted for mutagenesis indicated (*insets*, *stick* side chains). *B*, LnsA putative active site residues were selected based on conservation with LRAT family acyl transferases (3), and converted to Ala. Protein expression levels and TA-LP synthesis were determined by immunoblotting as in Figure 2. From *left* to *right*: GKM2702, TXM2829, TXM2830, TXM2831, TXM2832, TXM2833, TXM2834, TXM2835, TXM2836, and TXM2837). *C*, LnsB CPBP motif family residues were selected according to alignment (3), converted to Ala, and probed as in *panel B*. From *left* to *right*: GKM2702, TXM2878, TXM2867, TXM2868, TXM2869, TXM2870, TXM2890, TXM2871, TXM2872, and TXM2873. CPBP, CAAX protease and bacteriocin-processing; Lns, lipoprotein N-acylation system; LRAT, lecithin:retinol acyltransferase; TA-LP, tri-acylated bacterial lipoprotein.

LnsA is a member of the NlpC/P60 superfamily of enzymes (18), which encompasses four main families (P60-like, AcmB/LytN-like, YaeF/YiiX-like, and LRAT-like) (32). All families are thought to share an invariant Cys/His dyad that forms the covalent acyl-thioester substrate-enzyme intermediate, though the relative order of the dyad residues differs between canonical papain-like (P60-like and AcmB/LytN-like) and permuted papain-like (PPNE) (YaeF/YiiX-like and LRAT-like) families (18, 32). Based on sequence analysis, LnsA is a PPNE type acyl transferase and we previously inferred active site residues based on sequence conservation of His60 and Cys143 (3). Mapping of these residues into the heterodimeric Lns-LnsB Alphafold model places these two residues in proximity (Fig. 8*A*), and conversion of either of these residues to alanine abolished catalytic activity (Fig. 8*B*). Many NlpC/P60 superfamily members contain an essential signature tyrosine residue (33, 34, 35), and the corresponding residue in LnsA (Tyr129) which also colocates with His60/Cys143, was likewise essential for TA-LP biosynthesis. A third polar residue is thought to complete the active site triad in many PPNEs by orienting the essential His imidazole ring for deprotonation of the catalytic Cys (18, 32). Screening candidate polar residues (Gln72, Glu74, Cys61, Ser62) near the active site pocket, however, did not reveal an essential role for any of these residues. Notably, all three essential LnsA residues (His60, Cys143, and Tyr29) are within 3-4 A of the α-amino acceptor on the DA-LP ligand in the Protenix multimeric complex model (Fig. S3*C*).

We previously hypothesized that LnsB may not have a catalytic role, but rather be a substrate chaperone based on marginal sequence conservation both between *S. aureus* and *S. epidermidis* orthologs and with other CPBPs at key motifs positions (3). To test this, we mutated a series of residues in motif 1 (C167, Q168, R172), motif 2 (E196, Y200, Y201), and motif 4 (Q238, I242) to alanine. None of the mutations affected TA-LP synthesis (Fig. 8*C*), indicating CPBP motifs are not relevant to the catalytic function of LnsB.

### LnsA-LnsB utilizes the *SN*_1_-acyl chain of PG as the *N*-acyl donor

**Figure 9:**
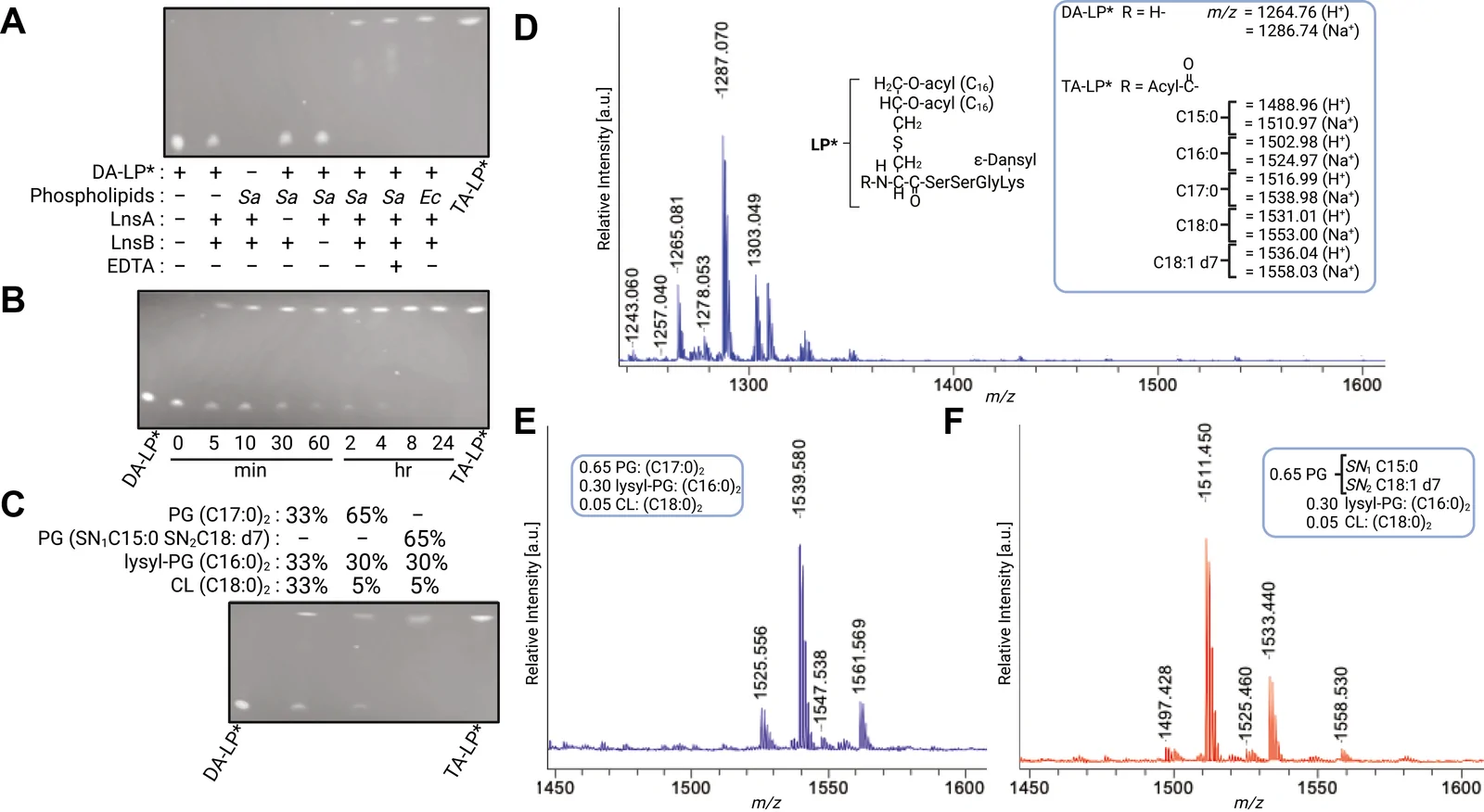
**Reconstitution of lipoprotein acylation requires both LnsA and LnsB and uses the *SN*_1_ acyl chain of phosphatidylglycerol as donor.***A*, recombinant LnsA and LnsB with total phospholipid extracts from either *S. aureus* (*Sa*) or *E. coli* (*Ec*) were added as indicated to fluorescently labeled DA-LP∗ lipopeptide substrate. Reactions were quenched and separated by TLC before being visualized under UV-light (365 nm). MALDI spectra for TA-LP∗ standard and TA-LP∗ produced using *S. aureus* total phospholipid extracts are shown in Fig. S4, *A* and *B*). DA-LP∗ = di-palmitoyl *S*-glyceryl-Cys-Ser-Ser-Gly-Lys-ε-dansyl peptide; TA-LP∗ = synthetic *N-*palmitoylated DA-LP∗ lipopeptide standard. *B*, replete lipoprotein acylation reactions were sampled over time for product formation as in *panel A*. *C*, reactions were assembled with various phospholipid mole fraction percentages of symmetrical C17:0 phosphatidylglycerol [PG(C17:0)_2_], asymmetric phosphatidylglycerol [PG(*SN*_1_ C15:0 *SN*_2_ C18:*d*7)], lysyl-phosphatidyl glycerol (lysyl-PG), and symmetric cardiolipin [CL(C18:0)_2_]. Reactions were separated by TLC and spots were scraped for lipopeptide elution. *D*, the MALDI spectra of the starting DA-LP∗ lipopeptide substrate was determined using α-CHCA matrix, with the expected peak masses for major protonated and sodiated TA-LP∗ products shown (*inset*). *E*, the spectra of TA-LP∗ arising from the indicated mix of phospholipids (*inset*), with the dominant peak having a mass consistent with C17:0 acyl chain transfer from PG. Similar spectra were obtained for 1:1:1 phospholipid mixtures (see Fig. S4*C*). *F*, reaction products using a phospholipid mixture with asymmetric PG donor (*inset*) yielded TA-LP∗ product mass peaks consistent with C15:0 acyl transfer originating from the *SN*_1_ position. DA-LP∗, di-palmitoyl S-glyceryl-Cys-Ser-Ser-Gly-Lys-ε-dansyl peptide; Lns, lipoprotein N-acylation system; PG, phosphatidylglycerol; TA-LP∗, *N*-palmitoyl di-palmitoyl *S*-glyceryl-Cys-Ser-Ser-Gly-Lys-ε-dansyl.

We cloned, overexpressed, and purified LnsA (lacking the signal peptide) and LnsB in *N*-dodecyl-β-D-maltoside (DDM) detergent micelles from *E. coli* to determine the phospholipid substrate preference. Reconstitution of the acylation system using a fluorescently-labeled DA-LP∗ model lipopeptide acceptor and a TLC assay to separate TA-LP product showed that both recombinant LnsA and LnsB are required, along with a phospholipid donor source (Fig. 9*A*). Once more, TA-LP formation was time dependent under replete reaction conditions (Fig. 9*B*). We initially utilized *S. aureus* total polar lipid extracts, whose membranes mainly contain a mixture of PG, lysyl-phosphatidylglycerol (lysyl-PG), and cardiolipin (CL) with saturated and branched fatty acids (predominantly C15:0 to C18:0) (36, 37, 38, 39). Mass spectrometry analysis of the TA-LP product demonstrated that a broad range of *N*-acyl chain lengths (C14:0 to C:20) could be incorporated (Fig. S4*B*), consistent with teh heterogeneous mass profiles of native lipoprotein extracts from *S. aureus* cells (3, 7). Total polar phospholipid extracts from *E. coli* could substitute for *S. aureus* phospholipids (Fig. 9*A*), suggesting either a common donor or LnsAB donor acyl chain substrate promiscuity. To further define the preferred phospholipid substrate, we mixed pure aliquots of the three candidate phospholipid donors where each had two identical acyl chains of unique chain length [(C17:0)_2_ PG, (C16:0)_2_ lysyl-PG, (C18:0)_2_ CL]. We tested both a 1:1:1 mol fraction ratio or a 0.65 PG:0.30 lysyl-PG:0.05 CL ratio (Fig. 9*C*), which is a more physiologically relevant *S. aureus* membrane composition (40). The TA-LP was TLC purified and analyzed by MALDI-TOF (Figs. 9, *D*, *E* and S4*C*). Regardless of the phospholipid donor pool composition, the dominant TA-LP product mass correlated with addition of a C17:0 acyl chain. Further fragmentation by MALDI-MS/MS confirmed that the C17:0 chain was attached to the *N* terminus (Fig. S5*C*). The data indicate PG is the preferred head group donor, but the acyl chain symmetry in (C17:0)_2_ PG prevented identification of which acyl chain is utilized. We thus set up a final reaction with an asymmetric PG donor in the pool so as to have unique masses for each PG acyl chain [0.65 *SN*_1_ C15:0 *SN*_2_ C18:0 *d*7 PG, 0.30 (C16:0)_2_ lysl-PG, 0.05 (C18:0)_2_ CL] and analyzed the TA-LP product as before. The MS (Fig. 9*F*) and MS/MS (Fig. S5*D*) spectra indicated addition of a C15:0 acyl chain to the α-amino group of the DA-LP∗ acceptor, identifying the *SN*_1_ position of PG as the source of fatty acids on the α-amino group of staphylococcal lipoproteins.

## Discussion

Acylation of the *N* terminus of bacterial lipoproteins converts DA-LP into TA-LP, reshaping how innate immunity recognizes and responds to different species of staphylococci (4). While initial genetic based studies clearly implicated the *lnsA* (SAOUHSC_00822) and *lnsB* (SAOUHSC_02761) genes being determinants for *N*-acylation in *S. aureus* (3), species level predictions on which lipoprotein chemotype is made, and hence the ensuing TLR2 response, is limited by a lack of a mechanistic understanding of LnsAB. The picture is further complicated by the seeming retention of syntenic CPBP/LnsB copies across the entire genera (Figs. 1 and S1). Bacterial gene similarity and shared synteny is generally regarded as a powerful predicter of conserved function (41). However, this does not seem to be the case for staphylococcal LnsB/CPBP candidates. Only upon MSA of CPBPs could syntenic LnsB orthologs from LnsA^+^ genomes be clearly coclustered (Fig. 1*C*). The syntenic copies of LnsB/CPBPs in LnsA^-^ genomes are likely involved in other cellular processes unrelated to lipoprotein biogenesis. The CPBP integral membrane protein family is proving itself to be quite functionally diverse, with LnsB joining the quorum sensing peptide protease MroQ/SAOUHSC_02256 (42, 43, 44), the peptidoglycan chain length release regulator LyrA/SpdC/SAOUHSC_02611 (45, 46), the regulator of the SpsB signal peptidase SpbR/SAOUHSC_00965 (47), and two more proteins of ill-defined function previously implicated in surface protein display (SdpA/SAOUHSC_01900 and SdpB/SAOUHSC_02587) (48). Many of these CPBPs participate in protein–protein recognition interactions within or at the extracellular membrane surface. With the notable exception of MroQ and akin to LnsB (Fig. 8*C*), most do not seem to be metalloproteases and the CPBP active site motifs do not contribute to function. The mosaic nature of the LnsB/CPBP locus across staphylococci likely reflects opportunistic recombination and genome rearrangement due to DNA level sequence similarity.

The presence/absence of LnsA thus far correlates with the TA-LP chemotype in staphylococci in a much more straightforward manner (Fig. 1). The *lnsA* gene is confined to a limited clade with conserved synteny, which also happens to harbor many of the more opportunistic pathogenic staphylococcal species (49, 50). The phylogenomic divide among environmental/pathogen associated is reminiscent of the Ac-LP (potent TLR2 agonist)/lyso-LP (weak TLR2 agonist) split observed in *Bacillus* species (8). Here we show LnsA is an exported protein with a canonical signal peptide that is cleaved after transit. We were initially puzzled by the DA-LP conversion to TA-LP when LnsA lacked an encoded signal peptide (Fig. 2*A*, *bottom panel*). Apparently, a portion of LnsA is still able to translocate to the outer leaflet, interact with LnsB, and access DA-LP substrate. Whether LnsA lacking signal peptide exits *via* transient membrane permeability or is cotransported with traditionally secreted proteins as has been proposed for moonlighting proteins that naturally lack noncanonical export signals (51), remains to be seen. It is clear, however, that efficient LnsA transport is signal peptide dependent (Fig. 5*C*) and LnsA remains tightly associated with the membrane even without a transmembrane α-helix or LnsB protein–protein anchoring interactions (Fig. 3*C*). These properties help explain why LnsA has not been classified as a secreted/SpsB dependent protein in *S. aureus* or *S. epidermidis* (52, 53, 54).

Alphafold/Protenix modeling (Fig. 4) with targeted disulfide crosslinking (Figs. 6 and 7) provided compelling evidence that LnsA and LnsB coassociate within cells. The interaction is defined in large part by loop 4 on LnsA (Fig. 6*A*), where even limited amino acid changes altered cross recognition between *S. aureus* and *S. epidermidis* orthologs (Figs. 6*D* and S2). Homodimeric LnsB crosslinking was readily induced with cysteine substitution in LnsB (Fig. 7*B*), suggesting a higher order complex exists in cells (Fig. S3). Confidence in a multimeric LnsAB complex was further enhanced by modeling results, which increased ipTM scores with DA-LP ligand when multimeric (tetrameric to hexameric, Table S3). Of note, the model positions the α-amino acceptor group within the experimentally determined LnsA catalytic acyl transfer center (His60/Cys143/Tyr129) (Figs. 8*B* and S3*C*). We could not identify an additional essential polar residue that is normally associated with NlpC/P60 enzymes (18, 32). In this regard, LnsA is an exception akin to LRATs where the equivalent polar residue was also not essential for catalysis (55). Attempts here to more precisely define LnsA-B complex stoichiometries by gel filtration or analytical ultracentrifugation were unsuccessful due to challenges with stability and heterogeneity, and will require further study.

Initial attempts to express LnsA in *E. coli* were hampered by extreme toxicity, likely resulting from problems with protein export and perturbation of membrane integrity. Removing the signal peptide and appending a large, hydrophilic *N*-terminal tag with SUMO helped reduce the toxicity and facilitate purification in the absence of exogenous detergents though the protein showed high propensity to aggregate consistent with a hydrophobic face. Both LnsA and LnsB were required for enzymatic activity (Fig. 9*A*), recapitulating our prior genetic based results showing both proteins are needed for TA-LP synthesis (3). Reconstitution allowed us to define the origin of the *N*-terminal acyl chain on TA-LP to the *SN*_1_-positon of PG (Fig. 9, *E* and *F*). In *S. aureus*, the acyl transferase PlsY acylates glycerol 3-phosphate at the *SN*_1_ position using acyl-phosphates while PlsC uses acyl-ACP as donor for the *SN*_2_ position to complete the synthesis of the phosphatidic acid pool that feeds phospholipid synthesis (56, 57). Unlike PlsY, PlsC is highly specific for C15:0 branched chain fatty acids (58, 59). Since LnsAB uses the *SN*_1_ acyl chain of PG, the acyl chain length diversity installed by PlsY should carry through to the α-amino terminus of lipoproteins provided LnsAB does not discriminate. This is entirely consistent with earlier mass spectrometry studies characterizing TA-LP from *S. aureus* showing highly heterogenous *N*-acyl group lipoprotein populations (3, 7, 60), and the incorporation of both C15:0 and C17:0 as seen here (Fig. 9, *E* and *F*).

## Experimental procedures

### Bacterial strains and growth conditions

Strains of *E. coli* were cultured in lysogeny broth (LB, 10 g tryptone, 10 g NaCl, 5 g yeast extract), while strains of *S. aureus* were grown in tryptic soy broth (TSB) supplemented with appropriate antibiotics for plasmid maintenance (erythromycin 5 μg/ml, chloramphenicol 10 μg/ml) and 1 mM Isopropyl β-D-1-thiogalactopyranoside (IPTG) for protein expression unless noted otherwise. All cultures were incubated at 37 °C with constant aeration (250 rpm). Construction methods for plasmids and strains used in this study can be found in the Supplementary Materials (Tables S1 and S2).

### Bioinformatics

The 16S rRNA sequences from annotated staphylococcal species were identified by pairwise BLAST using *S. aureus* subsp. *aureus* NCTC8325 as reference (61). Representative sequences from each species were collated and aligned using ClustalOmega (62), and phylogenomic trees visualized using iTOL (63). Top-scoring CPBP family protein sequences were identified in each genome through pairwise queries using LnsB from *S. aureus* SAOUHSC_02761 and *S. epidermidis* SE_2027 as reference with Biomni (64), followed by manual curation to include all syntenic staphylococcal CPBPs as well as all *S. aureus* paralogs from strain NCTC8325. Genome synteny with the LnsB locus from *S. aureus* (SAOUHSC_02761) was initially determined using SyntTax (65). Detailed synteny comparisons for select LnsB loci were performed by determining normalized BLAST bit scores for each ORF and identifying reciprocal best hits (threshold ≥ 10%) to define ortholog groups. ClustalOmega was used for multiple sequence alignments of staphylococcal CPBP proteins (62), and visualized using iTOL (63).

### Complementation with LnsA, LnsB, and DA-/TA-LP immunoblotting

The *S. aureus* strain TXM1576, which lacks both *lnsA* and *lnsB* as well as cross-reactive antibody binding cell envelope proteins *spa* and *sbi* (see Table S1), was used as plasmid host for functional complementation studies. Overnight cultures grown in TSB with appropriate antibiotic selection were diluted 1:100 v/v into 10 ml of TSB with antibiotics and IPTG for protein expression and grown to mid-exponential phase (OD_600nm_ ∼1.5). Cultures were harvested by centrifugation (5 min, 5000*g*), washed once with TBSE (20 mM Tris-HCl, pH 8.0, 130 mM NaCl, and 5 mM EDTA), and stored frozen at −80° C. Thawed pellets were resuspended in 800 μl of buffer (20 mM Tris-HCl, pH 8) supplemented with lysostaphin (25 μg/ml) and incubated at 37 °C for 40 min. An equal volume of 0.1 mm zirconia/silica beads was added, and cells disrupted at maximum speed on a homogenizer for 5 cycles of 30 s each (3800 rpm, Biospec). Samples were centrifuged at 3000*g* for 5 min at 4 °C to pellet beads and unbroken cells, and the ensuing supernatant further clarified by a second round of centrifugation (5000*g* for 10 min). Membranes were collected from the supernatant by ultracentrifugation at 100,000*g* for 1 h at 4 °C, the supernatant discarded, and the remaining pellets were directly resuspended in 100 μl of reducing 1X SDS page buffer. Samples were incubated at RT with shaking for 2 h, heated at 70 °C for 10 min, and loaded onto 12% Tris-glycine SDS PAGE gels for separation. Gels were transferred to nitrocellulose membranes for detection using either the α-6∗His, His-Tag mouse monoclonal antibody (catalog number 66005, Proteintech Group) or α-myc-Tag mouse monoclonal antibody (catalog number 2276S, Cell Signaling Technology) diluted 1:10,000 v/v in PBST for LnsA and LnsB, respectively. Bands were visualized using the goat anti-mouse IgG(H + L)-HRP conjugate (catalog number 15-035-003, Jackson ImmunoResearch Laboratories) diluted 1:5000 v/v in PBST and developed with Supersignal West Pico PLUS chemiluminescent substrate (Thermo Fisher Scientific). Membranes were stained with Ponceau S after developing to verify equivalent total protein loading. The ratio of DA-LP/TA-LP was assessed by coexpressing a 10-amino-acid *N*-terminal fragment of the SitC LP with the native signal peptide and a *C*-terminal strep tag epitope from plasmid pTXM759 (see Table S1). Cell pellets were harvested from aliquots (1.5 ml of culture, OD_600nm_ ∼1.5) of cultures grown as previously. Lipoprotein profiles were analyzed using 18% Tris-tricine SDS-PAGE electrophoresis and immunoblotting with StrepMAB-Classic HRP conjugate (catalog number 21509001, IBA Life Sciences) as described (3).

### Hemolysin secretion assay

Cultures harboring plasmids expressing variants of α-hly were transformed into *S. aureus* TXM1576 and grown to late mid-log (OD_600nm_ ∼1.5), diluted 1:100 v/v in fresh TSB, and spotted onto TSB-5% rabbit blood agar plates with erm for plasmid retention and IPTG for hemolysin expression. Plates were incubated at 37 °C for 24 h before imaging.

### Purification of LnsA from *S. aureus* for Edman degradation sequencing

Cultures of *S. aureus* strain TXM2686 expressing LnsA with a *C*-terminal His tag were grown at 37 °C in 500 ml TSB (with erm and IPTG) until OD_600nm_ ∼1.5. Cell pellets were collected (5000*g*, 10 min, 4 °C), washed with PBS, and resuspended in 10 ml PBS before being stored at −80 °C. The resuspension was thawed, immediately supplemented with 1 mM phenylmethylsulfonyl fluoride (PMSF) and 25 μg/ml of lysostaphin, and incubated at 37 °C for 40 min. Suspensions were broken by adding an equal volume of beads and bead beating as previously. Cells debris and beads were removed by centrifugation (5 min at 3000*g*), washed with buffer (20 mM Tris-HCl, pH = 8.0), and respun. The combined supernatants were clarified by centrifugation (5000*g* for 10 min), the membranes collected by ultracentrifugation (100,000*g* for 1 h at 4 °C), and stored at −80 °C. The membrane pellet was thoroughly resuspended in 700 μl of 100 mM sodium phosphate buffer (pH = 8.0) with 300 mM NaCl and 0.5% w/v Fos Choline 14 (F312S, Anatrace). After gently rotating at room temp for 1 h to solubilize LnsA-His, debris was removed by centrifugation (1 h at 20,000*g* at 4 °C). The supernatant was diluted with an equal volume of phosphate buffer lacking detergent to bring the concentration of detergent to 0.25%. The solution was incubated with 70 μl of His-tag Dynabeads (Invitrogen 10103D) for 10 min at RT, and then collected with a magnetic rack for 2 min before removing the supernatant by pipette. Beads were washed four times (100 mM sodium phosphate pH = 8, 300 mM NaCl, 0.05% Fos Choline 14), before elution with 1X nonreducing SDS-PAGE supplemented with 300 mM imidazole. Suspensions were boiled at 95 °C for 15 min, the beads removed by centrifugation, and eluted proteins were loaded in duplicate for separation by 12% SDS PAGE Tris-tricine electrophoresis. One sample set was transferred to a nitrocellulose membrane and developed by α-His immunoblotting as described previously, while a second was transferred to a PVDF membrane and stained with Coomassie blue. The immunoreactive band corresponding to LnsA-His was dissected and subject to 8 cycles of Edman degradation sequencing to determine the native *N* terminus (Iowa State University Protein Facility).

### Cellular localization of LnsA

Strains [GKM2702 (*lnsA*^+^*lnsB*^+^), TXM2859 (*lnsA*^*+*^ minus signal peptide, *lnsB*^*+*^), and TXM2878 (*lns* ^+^*lnsB*^−^)] were grown from 1:200 dilutions of overnight cultures in 25 ml TSB with selection antibiotics and 1 mM IPTG until ∼1.5 OD_600nm_. Cultures were pelleted at 3000*g* for 5 min, and supernatants were saved for trichloroacetic acid (TCA) protein precipitation of secreted proteins. The cell pellet was washed once in Tris buffered saline (20 mM Tris HCl, 130 mM NaCl, pH = 8.0; TBS), resuspended in 800 μl TBS with 1 mM PMSF, and lysed with lysostaphin followed by bead beating as described previously. After centrifugation (5000*g*, 10 min) to remove unbroken cells, the supernatant was ultracentrifuged (110,000*g*, 1 h at 4 °C) to separate the cytosolic supernatant from the membrane associated fraction. The membrane pellet was washed with TBS and recentrifuged, before being resuspended in 500 μl of 1 X SDS-PAGE loading buffer. Cytosolic and secreted protein fractions were precipitated by adding TCA to a final concentration of 20% w/v, incubating at RT for 10 min, and then collecting the protein precipitate by centrifugation (16,000*g*, 10 min 4 °C). Pellets were extensively washed five times with acetone, and then air-dried. Samples were resuspended directly in 500 μl of 1x SDS-PAGE loading buffer and neutralized with a minimal volume of concentrated Tris base. Samples were separated on a 12% SDS-PAGE gel, and probed for LnsA by immunoblotting as detailed previously.

### SCAM

SCAM was performed according to a modified protocol developed in *E. coli* (66). Strains expressing LnsA with cysteine introduced into three different positions and lacking all endogenously encoded cysteines (pGKM2703B, pGKM2703C, and pGKM2703D, see Table S1) were grown in 10 ml of TSB with 1 mM IPTG and 5 μg/ml of erm for plasmid maintenance at 37 °C until the OD_600nm_ reached ∼1.5. Cultures were pelleted (3000*g*, 5 min), resuspended in maleimide labeling buffer (50 mM Hepes, pH = 7.6, 150 mM NaCl, 0.1 mM MgSO_4_, 250 mM sucrose), and transferred to 1.7 ml Eppendorf microcentrifuge tubes. Cells were pelleted (3000*g*, 3 min), the supernatant removed, and then washed with 1 ml of labeling buffer. After centrifugation and removal of supernatant, cells were thoroughly resuspended by gently pipetting up and down in 250 μl of labeling buffer supplemented with 1 mM TCEP. Samples were further divided into four separate tubes, with 50 μl cell suspension aliquots in each. Blocking agents [*N*-ethyl maleimide, Thermo Fisher Scientific; AMS, Setareh Biotech; or two water only controls] were freshly prepared as 100 mM stock solutions in water and added in 2.5 μl aliquots to bring the concentration to ∼5 mM. Samples were incubated for 1 h at RT, before being quenched with 3 μl of 250 mM L-cysteine. After 5 min of incubation, cells were pelleted and washed with labeling buffer as previously to remove excess TCEP, blocking agents, and cysteine. The cell pellet was resuspended in 20 μl of digestion buffer (labeling buffer lacking sucrose and supplemented with 100 μg/ml lysostaphin and 50 U/ml of DNAse I) and incubated at 37 °C for 15 min to lyse cells. To the solution, 60 μl of lysis buffer (8 M urea, 1% SDS, 50 mM Hepes pH = 7.6) was added and samples vortexed. A 33 mM stock of mal-PEG (5000 Da) was made in 1:2 v/v dimethyl sulfoxide:water mixture, and 10 μl was added to each tube except for one of the water controls which received only the dimethyl sulfoxide:water solvent. Samples were labeled for 1 h at RT before being quenched with an equal volume of 2X SDS-PAGE loading buffer (50 mM Tris HCl, pH = 6.8, 6 M urea, 1% β-mercaptoethanol, 3% SDS, 10% glycerol, 0.1% bromophenol blue). Samples were incubated at 42 °C for 30 min before being analyzed by SDS-PAGE and immunoblotting for mass shifted His-tagged LnsA as described previously.

### Substituted cysteine disulfide crosslinking between LnsA and LnsB

Strains expressing various combinations of substituted cysteines introduced into pGKM2703A, which expresses LnsA and LnsB variants lacking all endogenous cysteines (see Table S1), were grown in 10 ml of TSB with 1 mM IPTG at 37 °C until the OD_600nm_ reached ∼1.5. Cells were collected by centrifugation (5000*g*, 5 min), washed with PBS, and resuspended in 1 ml of PBS with 100 μM DPS-4 crosslinker (4,4′-dithiopyridine, Thermo Fisher Scientific) to catalyze disulfide bond exchange. After a 30 min incubation at RT with gentle shaking, cells were collected (5000*g*, 3 min) and washed three times with TBSE before freezing pellets at −80 °C. Pellets were resuspended in 800 μl of 20 mM Tris-HCl, pH = 8, and broken with lysostaphin treatment and bead beating as previously detailed. Samples were centrifuged (3000*g*, 5 min at 4 °C) to pellet beads and unbroken cells, and the decanted supernatant clarified further (5000*g* for 10 min) before ultracentrifugation (100,000*g*, 1 h at 4 °C) to collect membranes. Membranes were resuspended in 100 μl of nonreducing 1 X SDS-PAGE loading buffer, incubated at RT for 2 h, separated by 12% SDS PAGE, and transferred to a nitrocellulose membrane for immunoblotting as described previously.

### Purification of recombinant LnsA protein

To purify recombinant LnsA with no signal peptide, pTXM1958 [pETDUET *N*-terminal 6X His-SMT3 SUMO tag-6X His-TEV site-LnsA (SOUHSC_00822), residues 32-189] was transformed into the *E. coli* BL21(DE3) derivative TXM2540 which lacks the endogenous lipoprotein *N*-terminal acyl transferase Lnt (8). Cultures were grown in 1 L of LB with carbenicillin (100 μg/ml) to an OD_600nm_∼0.5 at 37 °C, shifted to 16 °C, and protein expression induced with 1 mM IPTG. After shaking for 16 h, cells were collected by centrifugation (5000*g*, 10 min), resuspended in 35 ml of column buffer (20 mM Tris pH 8, 300 mM NaCl, 5 mM imidazole, 1 mM DTT, 10% glycerol, and 1 mM PMSF, and broken by three passes through a French pressure cell (14,000 lb/in^2^). Unbroken cells and debris were removed by centrifugation (18,000*g*, 20 min at 4 °C). Recombinant protein was then purified using HisPur cobalt resin (Thermo Fisher Scientific) according to the manufacturer’s instructions. Eluted protein was dialyzed overnight at 4 °C in dialysis buffer (20 mM Tris-HCl pH = 8.0, 300 mM NaCl, 1 mM DTT and 10% glycerol) containing GST tagged TEV protease (Nacalai USA Inc.) in manufacturer recommended amounts to cleave the SUMO tag. Protein was further purified using a 10/300 Superdex 200 gel filtration column (GE Healthcare) preequilibrated with dialysis buffer. Fractions containing purified LnsA protein were pooled, concentrated, and supplemented with glycerol to a final 20% concentration before being flash frozen in liquid nitrogen for storage at −80 °C.

### Purification of recombinant LnsB protein

The plasmid pGKM2137 [pET22b(+) *N*-terminal Strep tag-TEV site-LnsB (SAOUHSC_02761)] was transformed into the *E. coli* BL21(DE3) derivative TXM2540 and grown in 1 L of LB with carbenicillin (100 μg/ml) to an OD_600nm_∼0.5 at 37 °C. Expression was induced with1 mM IPTG, and incubated for a further 16 h with shaking at 16 °C. Cells were collected by centrifugation (5000*g*, 10 min), resuspended in 35 ml of column buffer (20 mM Tris pH = 8, 300 mM NaCl, 1 mM DTT, 10% glycerol, and 1 mM PMSF), and broken by three passes through a French pressure cell as previously. After centrifugation (18,000*g* for 20 min at 4 °C), the ensuing supernatant was clarified by ultracentrifugation (1 h at 100,000*g*, 4 °C) to collect membranes. Protein was extracted from the membranes by gentle shaking at RT for 1 h in solubilization buffer [1% DDM, 100 mM Tris-HCl, pH = 8.0, 150 mM NaCl, 1 mM DTT, 1 mM EDTA, 10% glycerol]. After a second round of ultracentrifugation, recombinant protein was purified on StrepActin XT column (IBA Lifesciences) according to manufacturer’s instructions using wash and elution buffers supplemented with 0.05% DDM. Eluted protein fractions were pooled and dialyzed overnight at 4 °C in dialysis buffer (0.05% DDM, 100 mM Tris HCl pH = 8.0, 150 mM NaCl, 1 mM DTT, 1 mM EDTA, and 10% glycerol) containing TEV protease to cleave the Strep tag. The TEV protease was removed by passing dialysate through a column containing glutathione resin, and the flow through was further purified using a 10/300 Superdex 200 gel filtration column in storage buffer (50 mM Tris HCl pH = 8, 150 mM NaCl, 1 mM DTT, 20% glycerol, and 0.05% DDM). Purified LnsB protein was concentrated by centrifugal filtration (Vivaspin, MWCO = 10 kDa), and flash-frozen in liquid nitrogen for storage at −80 °C.

### Reconstitution of LnsAB lipoprotein *N*-terminal acylation

Reactions were performed in a 20 μl total reaction volume with 1.2 μg of recombinant LnsA, 0.8 μg of recombinant LnsB, 25 μM of the fluorescently labeled lipopeptide DA-LP∗ substrate (di-palmitoyl *S*-glyceryl-Cys-Ser-Ser-Gly-Lys-ε-dansyl, Biosynth), and 25 μg of total phospholipids in a final reaction buffer containing 50 mM Tris-HCl pH = 8.0, 4% glycerol, 45 mM NaCl, 0.05% lauryl maltose neopentyl glycol (LMNG, Themo Scientific). Phospholipids were either extracted from *S. aureus* using a modified protocol (67) or purchased [*E. coli* Polar Lipid Extracts (Avanti, SKU: 100600P), (C17:0)_2_ PG (Avanti SKU: 830456), (C18:0)_2_ cardiolipin (Avanti SKU: 710334P), (C16:0)_2_ lysyl-phosphatidylglycerol (Avanti SKU: 840520P), C15:0-C18:1-*d*7- PG (Avanti SKU: 791640C)]. For *S. aureus* phospholipids, 1 L of TXM1576 was grown in TSB to late exponential phase (OD_600nm_ ∼3) at 37 °C. Cells were harvested by centrifugation (5000*g*, 10 min), washed with PBS, resuspended in 20 ml of water, frozen, and lyophilized to dryness. Powdered cells were extracted with 114 ml of a solvent mixture added in the sequence chloroform/methanol/water using volumes of 30/60/24 ml, respectively. Suspensions were stirred for 16 h at RT, transferred to a separatory funnel, and partitioned by adding 30 ml of chloroform and 30 ml of a 1% aqueous NaCl solution. After shaking, the lower chloroform phase was isolated and the volume reduced to ∼5 ml by rotary evaporation. To this solution, 50 ml of water was added and rotovapping continued to remove the remaining chloroform and reduce the volume to ∼10 ml. This aqueous solution was transferred to a tared vessel, frozen, and lyophilized to obtain the *S. aureus* total phospholipid extract used in this study. To make phospholipid mixtures with defined mole fractions from pure lipid stocks, single phospholipids dissolved in either chloroform or chloroform:methanol (2:1 v/v) were mixed together in desired ratios, and the bulk of organic solvents removed by a blowing nitrogen stream. Phospholipid films were then resuspended in water and placed in a sonicating water bath at 50 °C for 10 min before being frozen and lyophilized. Assembled enzyme reactions were typically incubated at 37 °C for 8 h unless indicated.

To extract and analyze the products, a TLC based assay was employed as has been described (8). Briefly, reactions were quenched by an equal volume of 70% ethanol (20 μl) followed by addition of 12 μl of chloroform. After vortexing, tubes were centrifuged (2 min at 13,000*g*), and the lower chloroform phase was spotted on Silica gel 60 TLC plates (Sigma-Aldrich) and developed in chloroform:methanol:water:acetic acid (65:25:4:1 v/v/v/v). A synthetic standard (*N*-palmitoyl di-palmitoyl *S*-glyceryl-Cys-Ser-Ser-Gly-Lys-ε-dansyl, Biosynth) was included on each TLC plate to monitor progress of reactions. Lipopeptide were visualized by UV light excitation (365 nm).

### MALDI-mass spectrometry

Fluorescent spots of interest from the TLC plate were scraped, mixed with 50 μl of chloroform:methanol (2:1 v/v), vortexed, and centrifuged (2 min at 16,000*g*). Supernatants were mixed 1:1 with a 10 mg/ml solution of α -cyano-4-hydroxycinnamic acid matrix and deposited onto a steel target plate. Mass spectra and MS/MS spectra in LIFT mode were collected using an Ultraflextreme (Bruker Daltonics) MALDI-TOF mass spectrometer.

## Data availability

All data described are contained within the article and in Supplementary Materials.

## Supporting information

This article contains supporting information (3, 8,25, 26, 28, 62, 64, 68, 69, 70, 71, 72, 73).

## Conflict of interest

The authors declare that they have no conflicts of interest with the contents of this article.
