# Supplementary material for: Reconstitution of the LnsAB lipoprotein N-acylation system complex from Staphylococcus aureus and characterization of substrate preference
Source: J Biol Chem. 2026 Aug 11;302(10):113434. doi: 10.1016/j.jbc.2026.113434 (PMC13559823; doi:10.1016/j.jbc.2026.113434)
Supplement: Supplementary file 1 [file mmc1.pdf]

SUPPOPRTING INFORMATION

Reconstitution of the LnsAB lipoprotein *N*-acylation system complex from *Staphylococcus aureus* and characterization of substrate preference

John H. Gardiner IV[a], Gloria Komazin[b] and Timothy C. Meredith[a,b]*

[a] The Huck Institutes of the Life Sciences, The Pennsylvania State University, University Park, Pennsylvania, USA 16802

[b] Department of Biochemistry and Molecular Biology, The Pennsylvania State University, University Park, Pennsylvania, USA 16802

* Address correspondence to Timothy C. Meredith.

Email: txm50@psu.edu

This PDF file includes:

Supplementary Methods
Supplementary Tables S1 and S3
Supplementary Figures S1 to S5

Supplementary Methods

Plasmid construction. The *lnsA* (SAOUHSC_00822) gene from *S. aureus* NCTC8325 was PCR amplified (primers TM2934-2121) and cloned into the cadmium inducible shuttle vector pCN59 (1) that had been digested with *EcoR*I/*Asc*I using the In-Fusion cloning kit (Takara Biosciences) to make pCN59-*lnsA*. The P_{pen}-*lacI* P_{spac} IPTG inducible promoter cassette was amplified with primers TM2947-2950 from pDR110 (2) and introduced into *Eco*RI/*Sph*I digested pCN59-*lnsA* to make pCN59 Pspac *lnsA*. A 6X *C*-terminal His tag on LnsA was introduced into pTXM1524, which contains *lnsB* (SAOUHSC_02761) *lnsA* (SAOUHSC_00822) cloned in tandem (3), by inverse PCR (primers GK2745-46). The pCN59 P_{spac} *lnsA* vector was digested with *Eco*RI/*Asc*I to receive the *lnsB lnsA* 6x His cassette (amplified from pTXM1524 with primers JG2977-TM2121). The c-myc tag was introduced into the *N*-terminus of *lnsB* by inverse PCR using this vector as template with primers TM3523-24 to make pTXM2684A (**Supplementary Table S1**). The resulting vector was the parent construct for other tagged LnsAB complementation vectors. To remove *lnsA* and make pTXM2684B, pTXM2684A was used as template for inverse PCR (JG2517-TM3748). The backbone of pTXM2684A was PCR amplified (primers TM3933-TM3930) to exclude *S. aureus lnsB* and introduce the upstream 5' sequence of *S. epidermidis lnsB* (SE_2727) with a c-myc tag. The PCR product was combined with the SE_2727 encoding PCR product (primers TM3931-32) amplified from *S. epidermidis* ATCC12228 genomic DNA to make pTXM2684C. A similar strategy was used to make pTXM2684D, with *S. epidermidis lnsA* (SE_0583) amplified (primers TM3934-35) and swapped for the *S. aureus* ortholog (primers GK2745-TM3936 amplified from pTXM2684A). To make the *S. epidermidis N*-terminal c-myc *lnsB lnsA C*-terminal 6X-His dual tagged vector pTXM2684E, pTXM2684C was used as template (primers GK2745-TM3937) and combined with the *S. epidermidis lnsA* PCR product. Syntenic CPBP ORFs from *S. carnosus* were amplified (SCA_1941 with primers TM3941-42 and SCA_1942 with primers TM3945-46) and cloned into the inverse PCR products of pTXM2684A using primers TM3939-40 and TM3943-44 to make pTXM2684F and pTXM2684G, respectively. Primers JG2776-77 with pTXM2684A as

template were used to introduce the A27I mutation in the signal peptide in pTXM2684H. The α-hemolysin (SAOUHSC_01121) signal peptide (*N*-terminal 26 amino acids) was amplified (JG2780-81) and cloned into the PCR amplified plasmid backbone from pTXM2684A using JG2778-JG3481 to exchange signal peptides and make pTXM2684I. The LnsA putative signal peptide (1-27 amino acids) was removed by inverse PCR (primers TM3858-60) using pTXM2684A as template to make pTXM2684J. The vector expressing LnsA 6X-His tag only was made by inverse PCR of pTXM2684A using primers JG3145-46 to remove *lnsB* in pTXM2684K. Primers TM4030-31 were used with pTXM2684D as template to install the *S. aureus* LnsA putative signal peptide (1-27 amino acids with primers TM4032-4033) and make pTXM2684L. Loop exchange constructs in *S. epidermidis* LnsA were constructed by assembling loop 3 (primers TM4034-35) and loop 4 (primers TM4036-37) inverse PCR products with pTXM2684D template to make pTXM2684M and pTXM2684N, respectively.

The pHly plasmid series expresses α-hemolysin (hly) *C*-terminal 6X-His tag and related variants under control of the P_{spac} promoter. To introduce α-hly, the entire gene was amplified from *S. aureus* NCTC8325 genome (primers JG2780-TM3887) and cloned into the inverse PCR product of pTXM2684A (primers JG3481-GK2745) to replace *lnsA* and append the *C*-terminal 6X-His tag. The *lnsB* ORF was then removed (primers JG3145-46) as done above, and a D39C mutation was introduced for future cysteine labeling studies by inverse PCR (primers TM3889-90) and complete the parent vector pHlyA (**Supplementary Table S1**). The native signal peptide (1-26 amino acids) was removed by inverse PCR of pHlyA using primers TM3888-3904 to make pHlyB or converted to AA26I (primers TM3926-27) to make pHlyC. The *S. aureus* LnsA putative signal peptide (1-27 amino acids) was amplified (primers TM3902-03) and introduced into the inverse PCR product (primers TM3546-TM3904) of pHlyA to make the *S. aureus* LnsA signal peptide-α-hly fusion encoded by pHlyD. The A27I allele was then introduced by plasmid PCR (primers TM3928-29) to make pHlyE.

All cysteine residues were converted to alanines in pTXM2684A by successive rounds of inverse PCR using primers targeting LnsB C167 and LnsA C61A C105A C143A (see **Supplementary Table S2** for primers) to make the parent plasmid pGKM2703A. Cysteine residues for maleimide labeling of LnsA were introduced by PCR amplification of pGKM2703A with primers TM3668-69 to make S32C in pGKM2703B, JG3479-84 to make G109C in pGKM2703C, and TM3710-11 to make V186C in pGKM2703D. The *lnsB* encoding gene was removed by inverse PCR of pGKM2703A with primers JG3145-46 to make pGKM2703E, which was then mutagenized as above to introduce S32C (pGKM2703F), G109C (pGKM2703G), and V186C (pGKM2703H). The A27I mutation was introduced into pGKM2703B with primers JG2777-TM3856 to make pGKM2703I. The α-hemolysin signal peptide (1-26 amino acids, JG2780-81) was cloned into the PCR amplified plasmid backbone of pGKM2703B (primers TM3857-JG3481) to form pGKM2703J, while the signal peptide was removed by inverse PCR (primers TM3859-60) to yield pGKM2703K. Targeted cysteines residues were likewise introduced by plasmid inverse PCR into the cysteine-less construct pGKM2703A to make LnsB D139C/M67C/I40C/V52C and LnsA Y163C/G56C/L165C (and combinations thereof) in pGKM2703L through pGKM2703V for cysteine disulfide crosslinking.

Putative active site LnsA and LnsB residues were converted to alanine by inverse PCR using pTXM2684A as template to make the pTXM2684-AX##A and pTXM2684-BX##A series, respectively (see **Supplementary Table S2** for corresponding primers).

Recombinant protein expression vectors were built in plasmids with T7 RNA polymerase inducible promoters. For LnsA, residues 32-189 were PCR amplified (primers TM2569-74) and cloned into a modified pET DUET based vector (lab stock) digested with *Nhe*I/*Hind*III. The construct pTXM1958 encodes LnsA with an *N*-terminal 6X-His SMT3 SUMO-6X His tag-TEV cut site replacing the native signal peptide. For LnsB, primers GK2769-TM2128 were used to amplify LnsB and cloned into a modified pET22b(+) based vector (lab stock) digested with *Nhe*I/*Xho*I.

The resulting vector was then subject to inverse plasmid PCR (primers GK2771-72) to introduce an *N*-terminal strep tag followed by a TEV cut site to make pGKM2137.

Strain Construction. All plasmids were introduced into *S. aureus* by electroporation (4). To delete *sbi* (SAOUHSC_02706) and *spa* (SAOUHSC_00069), in-frame deletion cassettes were assembled by PCR of ~1 kb of flanking DNA on each side (KK2176-77 P1/P2 with TM2178-79 P3/P4 and TM766-67 P1/P2 with TM768-69). Amplicons were assembled into the *E. coli*-*S. aureus* shuttle vector pKFC (5) digested with *Eco*RI/*Hind*III using In Fusion (Takara Biosciences). Markerless gene deletions were made by culture temperature cycling the gene deletion vectors in *S. aureus* and confirmed by PCR check (primers KK2180-81 or TM770-71) as has been described (6).

Table S1: Bacterial strains and plasmids

Strains/ plasmids	Relevant Genotype/Phenotype[1]	Source or reference
RN4220	S. *aureus* NCTC8325	Lab Stock
TM1500	ΔSAOUHSC_00822 (*lnsA*) ΔSAOUHSC_02761 (*lnsB*)	Ref. (3)
TXM1576	TXM1500 ΔSAOUHSC_02706 (*sbi*) ΔSAOUHSC_00069 (*spa*)	This study
TXM2686	TXM1576 (pTXM2684A); Erm^r	This study
GKM2702	TXM1576 (pTXM2684A) (pTXM759); Cat^r Erm^r	This study
TXM2829	TXM1576 (pTXM2684B) (pTXM759); Cat^r Erm^r	This study
TXM2874	TXM1576 (pTXM2684C) (pTXM759); Cat^r Erm^r	This study
TXM2875	TXM1576 (pTXM2684D) (pTXM759); Cat^r Erm^r	This study
TXM2876	TXM1576 (pTXM2684E) (pTXM759); Cat^r Erm^r	This study
TXM2879	TXM1576 (pTXM2684F) (pTXM759); Cat^r Erm^r	This study
TXM2880	TXM1576 (pTXM2684G) (pTXM759); Cat^r Erm^r	This study
TXM2855	TXM1576 (pTXM2684H) (pTXM759); Cat^r Erm^r	This study
TXM2857	TXM1576 (pTXM2684I) (pTXM759); Cat^r Erm^r	This study
TXM2859	TXM1576 (pTXM2684J) (pTXM759); Cat^r Erm^r	This study
TXM2878	TXM1576 (pTXM2684K) (pTXM759); Cat^r Erm^r	This study
JG2431	TXM1576 (pJG2424); Erm^r	This study
TXM2885	TXM1576 (pHlyA); Erm^r	This study
TXM2886	TXM1576 (pHlyB); Erm^r	This study
TXM2887	TXM1576 (pHlyC); Erm^r	This study
TXM2888	TXM1576 (pHlyD); Erm^r	This study
TXM2889	TXM1576 (pHlyE); Erm^r	This study
TXM2722	TXM1576 (pGKM2703A); Erm^r	This study
TXM2791	TXM1576 (pGKM2703B); Erm^r	This study
TXM2792	TXM1576 (pGKM2703C); Erm^r	This study
TXM2800	TXM1576 (pGKM2703D); Erm^r	This study
TXM2820	TXM1576 (pGKM2703E); Erm^r	This study
TXM2821	TXM1576 (pGKM2703F); Erm^r	This study
TXM2822	TXM1576 (pGKM2703G); Erm^r	This study
TXM2823	TXM1576 (pGKM2703H); Erm^r	This study
TXM2856	TXM1576 (pGKM2703I); Erm^r	This study
TXM2858	TXM1576 (pGKM2703J); Erm^r	This study
TXM2860	TXM1576 (pGKM2703K); Erm^r	This study
GKM2816	TXM1576 (pGKM2703L); Erm^r	This study
GKM2817	TXM1576 (pGKM2703M); Erm^r	This study
GKM2818	TXM1576 (pGKM2703N); Erm^r	This study
TXM2787	TXM1576 (pGKM2703O); Erm^r	This study
TXM2788	TXM1576 (pGKM2703P); Erm^r	This study
TXM2790	TXM1576 (pGKM2703Q); Erm^r	This study
TXM2797	TXM1576 (pGKM2703R); Erm^r	This study
TXM2798	TXM1576 (pGKM2703S); Erm^r	This study
TXM2927	TXM1576 (pTXM2684L) (pTXM759); Cat^r Erm^r	This study
TXM2928	TXM1576 (pTXM2684M) (pTXM759); Cat^r Erm^r	This study
TXM2929	TXM1576 (pTXM2684N) (pTXM759); Cat^r Erm^r	This study
TXM2925	TXM1576 (pGKM2703T); Erm^r	This study
TXM2777	TXM1576 (pGKM2703U); Erm^r	This study
TXM2727	TXM1576 (pGKM2703V); Erm^r	This study
TXM2830	TXM1576 (pTXM2684-LnsA-H60A) (pTXM759); Cat^r Erm^r	This study
TXM2831	TXM1576 (pTXM2684-LnsA-Y129A) (pTXM759); Cat^r Erm^r	This study
TXM2832	TXM1576 (pTXM2684-LnsA-C143A) (pTXM759); Cat^r Erm^r	This study

TXM2833	TXM1576 (pTXM2684-LnsA-Q72A) (pTXM759); Cat^r Erm^r	This study
TXM2834	TXM1576 (pTXM2684-LnsA-E74A) (pTXM759); Cat^r Erm^r	This study
TXM2835	TXM1576 (pTXM2684-LnsA-Q72AE74A) (pTXM759); Cat^r Erm^r	This study
TXM2836	TXM1576 (pTXM2684-LnsA-C61A) (pTXM759); Cat^r Erm^r	This study
TXM2837	TXM1576 (pTXM2684-LnsA-S62A) (pTXM759); Cat^r Erm^r	This study
TXM2867	TXM1576 (pTXM2684-LnsB-C167A) (pTXM759); Cat^r Erm^r	This study
TXM2868	TXM1576 (pTXM2684-LnsB-Q168A) (pTXM759); Cat^r Erm^r	This study
TXM2869	TXM1576 (pTXM2684-LnsB-R172A) (pTXM759); Cat^r Erm^r	This study
TXM2870	TXM1576 (pTXM2684-LnsB-E196A) (pTXM759); Cat^r Erm^r	This study
TXM2890	TXM1576 (pTXM2684-LnsB-Y200A) (pTXM759); Cat^r Erm^r	This study
TXM2871	TXM1576 (pTXM2684-LnsB-Y201A) (pTXM759); Cat^r Erm^r	This study
TXM2872	TXM1576 (pTXM2684-LnsB-Q238A) (pTXM759); Cat^r Erm^r	This study
TXM2873	TXM1576 (pTXM2684-LnsB-I242A) (pTXM759); Cat^r Erm^r	This study
TXM2540	*E. coli* BL21(DE3) *lpp::catR* (pTXM1026) *lnt::specR* by P1vir transduction; Kan^r Cat^r Spec^r	Ref. (7)
**Plasmids**		
pCN59	*S. aureus*-*E. coli* shuttle vector; Amp^r Cat^r	Ref. (1)
pTXM759	pLI50-P_{tuf} (Sa) SitC *N*-terminal 10 aa fragment-strep tag *blaz* transcriptional terminator; Cat^r	Ref. (3)
pJG2424	pCN59 with a P_{pen} *lacI* P_{spac} cassette and *EcoR*I/*Asc*I restriction sites replacing the P_{cadC} *luxAB* cassette; Cat^r	Lab stock
pTXM2684A	pCN59 P_{pen} *lacI* P_{spac} *N*-terminal c-myc *S. aureus* SAOUHSC_02761 (LnsB) and *C*-terminal 6X His tag *S. aureus* SAOUHSC_00822 (LnsA) *blaz* transcriptional terminator; Erm^r	This study
pTXM2684B	pCN59 P_{pen} *lacI* P_{spac} *N*-terminal c-myc *S. aureus* SAOUHSC_02761 (LnsB) *blaz* transcriptional terminator; Erm^r	This study
pTXM2684C	pCN59 P_{pen} *lacI* P_{spac} *N*-terminal c-myc *S. epidermidis* SE_2027 (LnsB) and *C*-terminal 6X His tag *S. aureus* SAOUHSC_00822 (LnsA) *blaz* transcriptional terminator; Erm^r	This study
pTXM2684D	pCN59 P_{pen} *lacI* P_{spac} *N*-terminal c-myc *S. aureus* SAOUHSC_02761 (LnsB) and *C*-terminal 6X His tag *S. epidermidis* SE_0583 (LnsA) *blaz* transcriptional terminator; Erm^r	This study
pTXM2684E	pCN59 P_{pen} *lacI* P_{spac} *N*-terminal c-myc *S. epidermidis* SE_2027 (LnsB) and *C*-terminal 6X His tag *S. epidermidis* SE_0583 (LnsA) *blaz* transcriptional terminator; Erm^r	This study
pTXM2684F	pCN59 P_{pen} *lacI* P_{spac} *N*-terminal c-myc *S. carnosus* SCA_1941 (CPBP family) and *C*-terminal 6X His tag *S. aureus* SAOUHSC_00822 (LnsA) *blaz* transcriptional terminator; Erm^r	This study
pTXM2684G	pCN59 P_{pen} *lacI* P_{spac} *N*-terminal c-myc *S. carnosus* SCA_1942 (CPBP family) and *C*-terminal 6X His tag *S. aureus* SAOUHSC_00822 (LnsA) *blaz* transcriptional terminator; Erm^r	This study
pTXM2684H	pCN59 P_{pen} *lacI* P_{spac} *N*-terminal c-myc *S. aureus* SAOUHSC_02761 (LnsB) and *C*-terminal 6X His tag *S. aureus* SAOUHSC_00822 A27I (LnsA) *blaz* transcriptional terminator; Erm^r	This study
pTXM2684I	pCN59 P_{pen} *lacI* P_{spac} *N*-terminal c-myc *S. aureus* SAOUHSC_02761 (LnsB) and *C*-terminal 6X His tag *S. aureus* SAOUHSC_00822 (LnsA) Δ1-27 aa replaced with α-hly (SAOUHSC_01121) signal peptide (1-26 aa) *blaz* transcriptional terminator; Erm^r	This study
pTXM2684J	pCN59 P_{pen} *lacI* P_{spac} *N*-terminal c-myc *S. aureus* SAOUHSC_02761 (LnsB) and *C*-terminal 6X His tag *S. aureus* SAOUHSC_00822 (LnsA) Δ1-27 aa No signal peptide *blaz* transcriptional terminator; Erm^r	This study

pTXM2684K	pCN59 P_{pen} *lacI* P_{spac} *C*-terminal 6X His tag *S. aureus* SAOUHSC_00822 (LnsA) *blaz* transcriptional terminator; Erm^r	This study
pTXM2684L	pCN59 P_{pen} *lacI* P_{spac} *N*-terminal c-myc *S. aureus* SAOUHSC_02761 (LnsB) and *C*-terminal 6X His tag *S. epidermidis* SE_0583 (LnsA) Δ1-28 aa replaced with LnsA *S. aureus* signal peptide (1-27 aa) *blaz* transcriptional terminator; Erm^r	This study
pTXM2684M	pCN59 P_{pen} *lacI* P_{spac} *N*-terminal c-myc *S. aureus* SAOUHSC_02761 (LnsB) and *C*-terminal 6X His tag *S. epidermidis* SE_0583 (LnsA) Δ120-134 aa replaced with LnsA *S. aureus* Loop 3 (125-139 aa) *blaz* transcriptional terminator; Erm^r	This study
pTXM2684N	pCN59 P_{pen} *lacI* P_{spac} *N*-terminal c-myc *S. aureus* SAOUHSC_02761 (LnsB) and *C*-terminal 6X His tag *S. epidermidis* SE_0583 (LnsA) Δ151-175 aa replaced with LnsA *S. aureus* Loop 4 (156-180 aa) *blaz* transcriptional terminator; Erm^r	This study
pHlyA	pCN59 P_{pen} *lacI* P_{spac} *C*-terminal 6X His tag *S. aureus* SAOUHSC_01121 (α-hemolysin) with D39C mutation *blaz* transcriptional terminator; Erm^r	This study
pHlyB	pCN59 P_{pen} *lacI* P_{spac} *C*-terminal 6X His tag *S. aureus* SAOUHSC_01121 (α-hemolysin) with D39C mutation and lacking the *N*-terminal signal peptide 1-26 amino acids *blaz* transcriptional terminator; Erm^r	This study
pHlyC	pCN59 P_{pen} *lacI* P_{spac} *C*-terminal 6X His tag *S. aureus* SAOUHSC_01121 (α-hemolysin) with D39C and AA26I mutations *blaz* transcriptional terminator; Erm^r	This study
pHlyD	pCN59 P_{pen} *lacI* P_{spac} *C*-terminal 6X His tag *S. aureus* SAOUHSC_01121 (α-hemolysin) with D39C mutation and the LnsA *N*-terminal 1-27 amino acid signal peptide replacing the native signal peptide *blaz* transcriptional terminator; Erm^r	This study
pHlyE	pCN59 P_{pen} *lacI* P_{spac} *C*-terminal 6X His tag *S. aureus* SAOUHSC_01121 (α-hemolysin) with D39C mutation and the LnsA *N*-terminal 27 amino acid A27I signal peptide replacing the native signal peptide *blaz* transcriptional terminator; Erm^r	This study
pGKM2703A	pCN59 P_{pen} *lacI* P_{spac} *N*-terminal c-myc tag *S. aureus* SAOUHSC_02761 (LnsB) C167A and *C*-terminal 6X His tag *S. aureus* SAOUHSC_00822 (LnsA) C61A C105A C143A *blaz* transcriptional terminator; Erm^r	This study
pGKM2703B	pCN59 P_{pen} *lacI* P_{spac} *N*-terminal c-myc tag *S. aureus* SAOUHSC_02761 (LnsB) C167A and *C*-terminal 6X His tag *S. aureus* SAOUHSC_00822 (LnsA) S32C C61A C105A C143A *blaz* transcriptional terminator; Erm^r	This study
pGKM2703C	pCN59 P_{pen} *lacI* P_{spac} *N*-terminal c -myc tag *S. aureus* SAOUHSC_02761 (LnsB) C167A and *C*-terminal 6X His tag *S. aureus* SAOUHSC_00822 (LnsA) G109C C61A C105A C143A *blaz* transcriptional terminator; Erm^r	This study
pGKM2703D	pCN59 P_{pen} *lacI* P_{spac} *N*-terminal c-myc *S. aureus* SAOUHSC_02761 (LnsB) C167A and *C*-terminal 6X His tag *S. aureus* SAOUHSC_00822 (LnsA) V186C C61A C105A C143A *blaz* transcriptional terminator; Erm^r	This study
pGKM2703E	pCN59 P_{pen} *lacI* P_{spac} *C*-terminal 6X His tag *S. aureus* SAOUHSC_00822 (LnsA) C61A C105A C143A *blaz* transcriptional terminator; Erm^r	This study

pGKM2703F	pCN59 P_{pen} *lacI* P_{spac} *C*-terminal 6X His tag *S. aureus* SAOUHSC_00822 (LnsA) S32C C61A C105A C143A *blaz* transcriptional terminator; Erm^r	This study
pGKM2703G	pCN59 P_{pen} *lacI* P_{spac} *C*-terminal 6X His tag *S. aureus* SAOUHSC_00822 (LnsA) G109C C61A C105A C143A *blaz* transcriptional terminator; Erm^r	This study
pGKM2703H	pCN59 P_{pen} *lacI* P_{spac} *C*-terminal 6X His tag *S. aureus* SAOUHSC_00822 (LnsA) V186C C61A C105A C143A *blaz* transcriptional terminator; Erm^r	This study
pGKM2703I	pCN59 P_{pen} *lacI* P_{spac} *N*-terminal c-myc *S. aureus* SAOUHSC_02761 (LnsB) C167A and *C*-terminal 6X His tag *S. aureus* SAOUHSC_00822 (LnsA) A27I S32C C61A C105A C143A *blaz* transcriptional terminator; Erm^r	This study
pGKM2703J	pCN59 P_{pen} *lacI* P_{spac} *N*-terminal c-myc *S. aureus* SAOUHSC_02761 (LnsB) C167A and *C*-terminal 6X His tag *S. aureus* SAOUHSC_00822 (LnsA) Δ1-27 aa replaced with α-hly signal peptide (1-26 aa) S32C C61A C105A C143A *blaz* transcriptional terminator; Erm^r	This study
pGKM2703K	pCN59 P_{pen} *lacI* P_{spac} *N*-terminal c-myc *S. aureus* SAOUHSC_02761 (LnsB) C167A and *C*-terminal 6X His tag *S. aureus* SAOUHSC_00822 (LnsA) Δ1-27 aa No signal peptide S32C C61A C105A C143A *blaz* transcriptional terminator; Erm^r	This study
pGKM2703L	pCN59 P_{pen} *lacI* P_{spac} *N*-terminal c-myc *S. aureus* SAOUHSC_02761 (LnsB) D139C C167A and *C*-terminal 6X His tag *S. aureus* SAOUHSC_00822 (LnsA) C61A C105A C143A *blaz* transcriptional terminator; Erm^r	This study
pGKM2703M	pCN59 P_{pen} *lacI* P_{spac} *N*-terminal c-myc *S. aureus* SAOUHSC_02761 (LnsB) D139C C167A and *C*-terminal 6X His tag *S. aureus* SAOUHSC_00822 (LnsA) Y163C C61A C105A C143A *blaz* transcriptional terminator; Erm^r	This study
pGKM2703N	pCN59 P_{pen} *lacI* P_{spac} *N*-terminal c-myc *S. aureus* SAOUHSC_02761 (LnsB) C167A and *C*-terminal 6X His tag *S. aureus* SAOUHSC_00822 (LnsA) Y163C C61A C105A C143A *blaz* transcriptional terminator; Erm^r	This study
pGKM2703O	pCN59 P_{pen} *lacI* P_{spac} *N*-terminal c-myc *S. aureus* SAOUHSC_02761 (LnsB) C167A and *C*-terminal 6X His tag *S. aureus* SAOUHSC_00822 (LnsA) L165C C61A C105A C143A *blaz* transcriptional terminator; Erm^r	This study
pGKM2703P	pCN59 P_{pen} *lacI* P_{spac} *N*-terminal c-myc *S. aureus* SAOUHSC_02761 (LnsB) I40C C167A and *C*-terminal 6X His tag *S. aureus* SAOUHSC_00822 (LnsA) C61A C105A C143A *blaz* transcriptional terminator; Erm^r	This study
pGKM2703Q	pCN59 P_{pen} *lacI* P_{spac} *N*-terminal c-myc *S. aureus* SAOUHSC_02761 (LnsB) M67C C167A and *C*-terminal 6X His tag *S. aureus* SAOUHSC_00822 (LnsA) C61A C105A C143A *blaz* transcriptional terminator; Erm^r	This study
pGKM2703R	pCN59 P_{pen} *lacI* P_{spac} *N*-terminal c-myc *S. aureus* SAOUHSC_02761 (LnsB) I40C C167A and *C*-terminal 6X His tag *S. aureus* SAOUHSC_00822 (LnsA) L165C C61A C105A C143A *blaz* transcriptional terminator; Erm^r	This study
pGKM2703S	pCN59 P_{pen} *lacI* P_{spac} *N*-terminal c-myc *S. aureus* SAOUHSC_02761 (LnsB) M67C C167A and *C*-terminal 6X His tag *S. aureus* SAOUHSC_00822 (LnsA) L165C C61A C105A C143A *blaz* transcriptional terminator; Erm^r	This study

pGKM2703T	pCN59 P_{pen} *lacI* P_{spac} *N*-terminal c-myc *S. aureus* SAOUHSC_02761 (LnsB) C167A and *C*-terminal 6X His tag *S. aureus* SAOUHSC_00822 (LnsA) G56C C61A C105A C143A *blaz* transcriptional terminator; Erm^r	This study
pGKM2703U	pCN59 P_{pen} *lacI* P_{spac} *N*-terminal c-myc *S. aureus* SAOUHSC_02761 (LnsB) V52C C167A and *C*-terminal 6X His tag *S. aureus* SAOUHSC_00822 (LnsA) C61A C105A C143A *blaz* transcriptional terminator; Erm^r	This study
pGKM2703V	pCN59 P_{pen} *lacI* P_{spac} *N*-terminal c-myc *S. aureus* SAOUHSC_02761 (LnsB) V52C C167A and *C*-terminal 6X His tag *S. aureus* SAOUHSC_00822 (LnsA) G56C C61A C105A C143A *blaz* transcriptional terminator; Erm^r	This study
pTXM2684-LnsA-H60A	pCN59 P_{pen} *lacI* P_{spac} *N*-terminal c-myc *S. aureus* SAOUHSC_02761 (LnsB) and *C*-terminal 6X His tag *S. aureus* SAOUHSC_00822 (LnsA) H60A *blaz* transcriptional terminator; Erm^r	This study
pTXM2684-LnsA-Y129A	pCN59 P_{pen} *lacI* P_{spac} *N*-terminal c-myc *S. aureus* SAOUHSC_02761 (LnsB) and *C*-terminal 6X His tag *S. aureus* SAOUHSC_00822 (LnsA) Y129A *blaz* transcriptional terminator; Erm^r	This study
pTXM2684-LnsA-C143A	pCN59 P_{pen} *lacI* P_{spac} *N*-terminal c-myc *S. aureus* SAOUHSC_02761 (LnsB) and *C*-terminal 6X His tag *S. aureus* SAOUHSC_00822 (LnsA) C143A *blaz* transcriptional terminator; Erm^r	This study
pTXM2684-LnsA-Q72A	pCN59 P_{pen} *lacI* P_{spac} *N*-terminal c-myc *S. aureus* SAOUHSC_02761 (LnsB) and *C*-terminal 6X His tag *S. aureus* SAOUHSC_00822 (LnsA) Q72A *blaz* transcriptional terminator; Erm^r	This study
pTXM2684-LnsA-E74A	pCN59 P_{pen} *lacI* P_{spac} *N*-terminal c-myc *S. aureus* SAOUHSC_02761 (LnsB) and *C*-terminal 6X His tag *S. aureus* SAOUHSC_00822 (LnsA) E74A *blaz* transcriptional terminator; Erm^r	This study
pTXM2684-LnsA-Q72AE74A	pCN59 P_{pen} *lacI* P_{spac} *N*-terminal c-myc *S. aureus* SAOUHSC_02761 (LnsB) and *C*-terminal 6X His tag *S. aureus* SAOUHSC_00822 (LnsA) Q72A E74A *blaz* transcriptional terminator; Erm^r	This study
pTXM2684-LnsA-C61A	pCN59 P_{pen} *lacI* P_{spac} *N*-terminal c-myc *S. aureus* SAOUHSC_02761 (LnsB) and *C*-terminal 6X His tag *S. aureus* SAOUHSC_00822 (LnsA) C61A *blaz* transcriptional terminator; Erm^r	This study
pTXM2684-LnsA-S62A	pCN59 P_{pen} *lacI* P_{spac} *N*-terminal c-myc *S. aureus* SAOUHSC_02761 (LnsB) and *C*-terminal 6X His tag *S. aureus* SAOUHSC_00822 (LnsA) S62A *blaz* transcriptional terminator; Erm^r	This study
pTXM2684-LnsB-C167A	pCN59 P_{pen} *lacI* P_{spac} *N*-terminal c-myc *S. aureus* SAOUHSC_02761 (LnsB) C167A and *C*-terminal 6X His tag *S. aureus* SAOUHSC_00822 (LnsA) *blaz* transcriptional terminator; Erm^r	This study
pTXM2684-LnsB-Q168A	pCN59 P_{pen} *lacI* P_{spac} *N*-terminal c-myc *S. aureus* SAOUHSC_02761 (LnsB) Q168A and *C*-terminal 6X His tag *S. aureus* SAOUHSC_00822 (LnsA) *blaz* transcriptional terminator; Erm^r	This study
pTXM2684-LnsB-R172A	pCN59 P_{pen} *lacI* P_{spac} *N*-terminal c-myc *S. aureus* SAOUHSC_02761 (LnsB) R172A and *C*-terminal 6X His tag *S. aureus* SAOUHSC_00822 (LnsA) *blaz* transcriptional terminator; Erm^r	This study
pTXM2684-LnsB-E196A	pCN59 P_{pen} *lacI* P_{spac} *N*-terminal c-myc *S. aureus* SAOUHSC_02761 (LnsB) E196A and *C*-terminal 6X His tag *S. aureus* SAOUHSC_00822 (LnsA) *blaz* transcriptional terminator; Erm^r	This study
pTXM2684-LnsB-Y200A	pCN59 P_{pen} *lacI* P_{spac} *N*-terminal c-myc *S. aureus* SAOUHSC_02761 (LnsB) Y200A and *C*-terminal 6X His tag *S. aureus* SAOUHSC_00822 (LnsA) *blaz* transcriptional terminator; Erm^r	This study

pTXM2684-LnsB-Y201A	pCN59 P_{pen} *lacI* P_{spac} *N*-terminal c-myc *S. aureus* SAOUHSC_02761 (LnsB) Y201A and *C*-terminal 6X His tag *S. aureus* SAOUHSC_00822 (LnsA) *blaz* transcriptional terminator; Erm^r	This study
pTXM2684-LnsB-Q238A	pCN59 P_{pen} *lacI* P_{spac} *N*-terminal c-myc *S. aureus* SAOUHSC_02761 (LnsB) Q238A and *C*-terminal 6X His tag *S. aureus* SAOUHSC_00822 (LnsA) *blaz* transcriptional terminator; Erm^r	This study
pTXM2684-LnsB-I242A	pCN59 P_{pen} *lacI* P_{spac} *N*-terminal c-myc *S. aureus* SAOUHSC_02761 (LnsB) I242A and *C*-terminal 6X His tag *S. aureus* SAOUHSC_00822 (LnsA) *blaz* transcriptional terminator; Erm^r	This study
pTXM1958	pETDuet-*N*-6X His tag-SMT3 SUMO-6X His tag-TEV- LnsA expression vector with T7 RNA polymerase promoter inducible by IPTG encoding LnsA (SAOUHSC_00822) residues 32-189 downstream of tag; final product post TEV treatment contains an extra 3 amino acids (SAS) appended to *N*-terminus after tag proteolysis; $Carb^r$	This study
pGKM2137	pET22-*N*-Strep tag-TEV site-LnsB Expression vector with T7 RNA polymerase promoter inducible by IPTG encoding LnsB (SAOUHSC_002761) residues 2-249 resulting in an additional Ser appended to *N*-terminus after tag proteolysis; $Carb^r$	This study
pKFC-*spa* KO	Shuttle vector with temperature sensitive replication origin with markerless deletion cassette for *spa* (SAOUHSC_00069); $Carb^r$, Cat^r	This study
pKFC- *sbi* KO	Shuttle vector with temperature sensitive replication origin with markerless deletion cassette for *sbi* (SAOUHSC_02706); $Carb^r$, Cat^r	This study

[1] Erm^r- erythromycin resistant; Cat^r- chloramphenicol resistant; $Carb^r$- carbenicillin; Kan^r- kanamycin.

Table S2: Primers used in this study

Primer Name	Primer Sequence
TM766- spa KO P1	GACGGCCAGTGAATTGCCTGAAGTCGATATGAC
TM767- spa KO P2	GCAATACCTACACCTAGTTTACG
TM768- spa KO P3	AGGTGTAGGTATTGCGACGTCGTCGCGAAC
TM769- spa KO P4	TGATTACGCCAAGCTGTATTAGTATGGAGTGC
TM770- spa KO check for	GTGCATATACAAGGAGATAAAACAATG
TM771- spa KO check rev	GAAATTGTCTTTGCTTGGAGTCCGTTC
KK2176 - sbi KO P1	GACGGCCAGTGAATTGGATACCTACAAACATAATGAC
KK2177 - sbi KO P2	ATATTTATTTTTCATGTGTATTCCCTTTC
KK2178 - sbi KO P3	ATGAAAAATAAATATTCATTCTGGAAATAATCAATC
KK2179 - sbi KO P4	TGATTACGCCAAGCTGAGTAGTTGAATGAGCCAC
KK2180 - sbi KO upcheck	GAACAAACGAGGTGCATTTTG
KK2181 - sbi KO downcheck	CACCTTTAGAGTTCTGACTTTCTAC
TM2947- PpenlacI SphI	GAGCTGGCGGCCGCTGCATGCGGTCTGATCGAAATAGTA
TM2950- lacI Sa	TGTTGAAGAAGAATTCTAGCTGTCGACTAAGC
TM2934- InsA 822 Pcad for EcoRI	TACCGAGCTCGAATTCTTCTTCAACAAGGAGC
TM2121- SA822 pCN59 rev AscI	ATTAGAATAGGCGCGCCCTATTTGATGAAACTGTG
GK2745- InsA-6His-For pCN59	GTGGCGGTCATCATCACCATCACCACTAACACAGTTTCAT CAAATAG
GK2746- InsA-6His-Rev pCN59	GATGATGACCGCCACCATAAGCTTTAACAAGCC
JG2977 - SaLnsB Pspac EcoRI Fw	TCGACAGCTAGAATTCAACTATTTAAGAGGT
TM3523- InsB Nterm cmyc for	GATTTCGGAAGAAGATTTAAATACAAATAAACATTAC
TM3524- InsB Nterm cmyc rev	TCTTCTTCCGAAATCAATTTTTGTTCTGACATAGTATACCTC
JG2517- Pspac myc tag InsB only rev	AAGCTCCTTGCTCTGACCATTTTAC
TM3748- Pspac myc tag InsB only for	CAGAGCAAGGAGCTTCACAGTTTCATCAAATAG
TM3933- InsB Se for fus plas for	CAACTTTTAAATCTCGGTCAGAGCAAGGAGC
TM3930- InsB Se myc rev plas rev	CAATTTTTGTTCATCCATATTGTCCCTCTTCTCGCATAGAAT TCTAGCTGTCGACTAAGC
TM3931- InsB Se myc gene for	GATGAACAAAAATTGATTTCGGAAGAAGATTTAATTAAACA ATCTTCAGAG
TM3932- InsB Se gene rev	GAGATTTAAAAGTTGGTATACTTAC
TM3934- InsA Se gene for	CTAGATTATATTTGAAGTAAATCAATTATTTTTGC
TM3935- InsA Se gene rev	GATGATGACCGCCACCATATGTTTTTACTAATTTTAATTTAT ATTCATCTG
TM3936- InsA Se plas rev	TCAAATATAATCTAGCCATTTTACATGAAAATATGC
TM3937- InsAB Se gene fusion	TCAAATATAATCTAGAGATTTAAAAGTTGGTATACTTAC
TM3939- SCA1941 plas for	CAACTGTTTCATTAAACTGGGTCAGAGCAAGGAGC
TM3940- SCA1941 plas rev	CAATTTTTGTTCCATGATGTTCACCTCTTGATGTGAATTCTA GCTGTCGACTAAGC
TM3941- SCA1941 gene for	ATGGAACAAAAATTGATTTCGGAAGAAGATTTAATAAATAA CCCATTCGATTC
TM3942- SCA1941 gene rev	TTAATGAAACAGTTGTTGATAGATG
TM3943- SCA1942 plas for	CTTAATTAAAAAATAACGGTCAGAGCAAGGAGC
TM3944- SCA1942 plas rev	CAATTTTTGTTCCGACATGTTTTCACCTCTATTATCGAATTC TAGCTGTCGACTAAGC

TM3945- SCA1942 gene for	TCGGAACAAAAATTGATTTCGGAAGAAGATTTAGATCAATTTACAAATAAAGAAC
TM3946- SCA1942 gene rev	TATTTTTTAATTAAGATATTGAACTATAATCG
JG2776 - InsA A27I Fw	CAATTATCGATAATAAGTATTCAGA
JG2777 - InsA A27I Rv	TTATCGATAATTGTTTTTGGTGA
JG2780 - InsA ahemolysin SP Fw	GCTTTTAAATGAAAACACGTATAGTCAG
JG2781 - InsA ahemolysin SP Rv	TTATTATCTGCGGCATTAGCGAC
JG3481 - InsA ahemo SP plas Rv	GTTTTCATTTAAAAGCTCCTTGC
JG2778 - InsA ahemo SP plas Fw	ATGCCGCAGATAATAAGTATTCA
TM3858- InsA no sp for	GGAGCTTTTAAATGGATAATAAGTATTCAG
TM3860- InsA no sp rev	CCATTTAAAAGCTCCTTGCTCTG
JG3145 - InsA only Pspac Fw	TAGAATTCAATGGTCAGAGCAAG
JG3146 - InsA only Pspac Rv	TGACCATTGAATTCTAGCTGTCG
TM4030- Se SP plas for	AATGATAATAAACGTTTTCAGC
TM4031- Se SP plas rev	CATATATTTAAAGAAGCTCCTTG
TM4032- Se SP fus for	TTCTTTAAATATATGAACTATATTAAACGAACG
TM4033- Se SP fus rev	ACGTTTATTATCATTTGCAATTGTTTTTGGTGAG
TM4034- Se InsA loop3 swap for	GTTACATTAAATTTGAACAGCAAAAAATTTACATATTGCACTA
TM4035- Se InsA loop3 swap rev	CAAATTTAATGTAACTAGATAACGATGATTTGTATTCTCATAATGTTTC
TM4036- Se InsA loop4 swap for	ATATTATTTCACCCTATGCAATTAAAGATAATTTTATAGATCCATATAAATTAAAATTAG
TM4037- Se InsA loop4 swap rev	AGGGTGAAATAATATGTAGTCCATAACTTTTAACTGATTTTTCACTTACGCCGTATTTAT
JG2780 - InsA Signal Peptide Fw	GCTTTTAAATGAAAACACGTATAGTCAG
TM3887- ahemolysin rev	GATGATGACCGCCACCATTTGTCATTTCTTCTTTTTCC
TM3889- ahemolysin D39C rev	ATTGCTTCCAATACATGTAGTACCGG
TM3890- ahemolysin D39C for	TGTATTGGAAGCAATACTACAG
TM3888- ahemolysin no sp rev	AATATCAGAATCTGCCATTTAAAAGCTCCTTGCTC
TM3904- ahemolysin no sp for	GCAGATTCTGATATTAATATTAAAACCG
TM3926- ahemolysin AA26I for	AATATTGATTCTGATATTAATATTAAAACCG
TM3927- ahemolysin AA26I rev	ATCAGAATCAATATTAGCGACAGGATTCATTA
TM3902- InsA SP hemolysin fus for	AGGAGCTTTTAAATGAACTATATTAAACG
TM3903- InsA SP hemolysin fus rev	AATATCAGAATCTGCTGCAATTGTTTTTGGTG
TM3546- pCN59 InsAplas RBS rev	CATTTAAAAGCTCCTTGCTCTGACC
TM3928- InsASPheme A27I for	CCAAAAACAATTATTGATTCTGATATTAATATTAAAACC
TM3929- InsASPhemeA271I rev	AATAATTGTTTTTGGTGAGTTTATTGG
JG2782 - InsB C167A Fw	TGATTGCACAACTTATTTTCCGT
JG2783 - InsB C167A Rv	AGTTGTGCAATCAAAGTATTTAATAT
JG2803 - InsA C61A Fw	GTCATGCAAGTATCGCGATTGAT
JG2804 - InsA C61A Rv	ATACTTGCATGACCAAAAAATCC
JG3161 - InsA C105A Fw	TATCGTGCAAGCTATCCTGGTGCAGGTAAA
JG3162 - InsA C105A Rv	ATAGCTTGCACGATAAACTTTCATCCAATC
JG3172 - InsA C143A Fw	ACGTATGCAACTAAAATTATCTACCAAGCT
JG3173 - InsA C143A Rv	TTTAGTTGCATACGTAAATTTTTTGCTGTT
TM3668 - InsA S32C for SCAM	TGTGAAATTCAAGATGACAAATTCC

TM3669 - InsA S32C rev SCAM	ATCTTGAATTTCACAATACTTATTATCTGC
JG3479 - InsA G109C for SCAM	ATCCTTGCGCAGGTAAAAAAGCG
JG3484 - InsA G109C rev SCAM	CCTGCGCAAGGATAGCTTGCACG
TM3710 - InsA V186C for SCAM	TGTAAAGCTTATGGTGGCGGTC
TM3711 - InsA V186C rev SCAM	ACCATAAGCTTTACAAAGCCTTAGTTTGTATGG
TM3856 - InsA S32C A27I for	CAATTATCGATAATAAGTATTGTGA
TM3857 - InsA S32C hemolysin	ATGCCGCAGATAATAAGTATTGT
TM3859 - InsA no sp S32C for	GGAGCTTTTAAATGGATAATAAGTATTGTG
JG3372 - InsB D139C Fw	TCTTTTGTATTTACCCGCTCATT
JG3373 - InsB D139C Rv	TAAATACAAAAGACAGTAAAGGA
JG3370 - InsA-Y163C Fw	AAAGTTGTGGACTACATATTATT
JG3371 - InsA-Y163C Rv	AGTCCACAACTTTTAACTGATTT
TM3670 - InsA L165C for	TGTCATATTATTTCACCCTATGC
TM3671 - InsA L165C rev	TGAAATAATATGACATCCATAACTTTTAACTG
TM3672 - InsB M67C for	TGTAACATTAGCTATTTACTCGCAG
TM3673 - InsB M67C rev	ATAGCTAATGTTACATGAAATAGGAAAAGAGAAAC
TM3676 - InsB I40C for	TGTATTATCGTCTTTTTAACTTTTGGG
TM3677 - InsB I40C rev	AAAGACGATAATACACGGCAAGATGTTAACC
GK3491 - InsB V52C for	TGTTTCACTCAACAAGCTCCGTGG
GK3492 - InsB V52C rev	TTGTTGAGTGAAACAAGCTACTAACCCAAAAG
GK3516 - InsA G56C C61A for	TGTTTTTTTGGTCATGCAAGTATCGCG
GK3494 - InsA G56C rev	ATGACCAAAAAAACACCACATGACAGGGCCT
JG2583 - InsA H60A Fw	GATTTTTTGGTGCATGTAGTATCGCGATT
JG2584 - InsA H60A Rv	ACTACATGCACCAAAAAATCCCCACA
TM3799 - InsA Y129 for	GCACTAGTTACATTAAATTTGAACAGC
TM3800 - InsA Y129 rev	TAATGTAACTAGTGCACGATGATTTGTATTCTC
JG3172 - InsA C143A Fw	ACGTATGCAACTAAAATTATCTACCAAGCT
JG3173 - InsA C143A Rv	TTTAGTTGCATACGTAAATTTTTTGCTGTT
JG2585 - InsA Q72A Fw	ATTTTAGCAATTGAGGGGCCTGGC
JG2586 - InsA Q72A Rv	CCCTCAATTGCTAAAATCGTTTTATCATC
JG2587 - InsA E74A Fw	CAAATTGCAGGGCCTGGCGACAAA
JG2588 - InsA E74A Rv	AGGCCCTGCAATTTGTAAAATCGT
JG2631 - InsA Q72A E74A Fw	ATTTTAGCAATTGCAGGGCCTGGCGACAAA
JG2632 - InsA Q72A E74A Rv	CGCCAGGCCCTGCAATTGCTAAAATCGTTTTATC
JG2803 - InsA C61A Fw	GTCATGCAAGTATCGCGATTGAT
JG2804 - InsA C61A Rv	ATACTTGCATGACCAAAAAATCC
JG2805 - InsA S62A Fw	ATTGTGCAATCGCGATTGATGAT
JG2806 - InsA S62A Rv	GCGATTGCACAATGACCAAAAAA
JG2782 - InsB C167A Fw	TGATTGCACAACTTATTTTCCGT
JG2783 - InsB C167A Rv	AGTTGTGCAATCAAAGTATTTAATAT
JG2784 - InsB Q168A Fw	TTTGTGCACTTATTTTCCGTCAT
JG2785 - InsB Q168A Rv	ATAAGTGCACAAATCAAAGTATTTAA

JG2786 - InsB R172A Fw	TTTTCGCACATATTTTAATTCAAGA
JG2787 - InsB R172A Rv	ATATGTGCGAAAATAAGTTGACA
JG2788 - InsB E196A Fw	TCCTTGCAACTCTGTTCTACTAT
JG2789 - InsB E196A Rv	AGAGTTGCAAGGATAATGGACAA
TM3962 - InsB Y200A for	CTGTTCGCATATCCATACATTAATAC
TM3963 - InsB Y200A rev	ATGGATATGCGAACAGAGTTTCAAGG
JG2790 - InsB Y201A Fw	TCTACGCACCATACATTAATACATG
JG2791 - InsB Y201A Rv	TATGGTGCGTAGAACAGAGTTTC
JG2792 - InsB Q238A Fw	TCTATGCATTAGCAGTTATACTC
JG2793 - InsB Q238A Rv	GCTAATGCATAGAAATAGCTCAC
JG2794 - InsB I242A Fw	CAGTTGCACTCGTTTTGCATATT
JG2795 - InsB I242A Rv	ACGAGTGCAACTGCTAATTGATA
TM2569- LnsA trunc for NheI	CTTCCAATCCGCTAGCTCAGAAATTCAAGATG
TM2574- LnsA rev HindIII	ACCAGACTCGAAGCTTAATAAGCTTTAACAAGC
GK 2771 - StrepTag TEV LnsB For	GCAGTTCGAAAAAGAGAACCTGTACTTCCAATCCTCAAATACAAATAAACATTACATAG
GK2772 - StrepTag TEV LnsB Rev	TCTTTTTCGAACTGCGGGTGGCTCCATGCGCTCATATGTATATCTCCTTCTTAAAG
GK2769 - pET22 LnsB NheI for	CTTCCAATCCGCTAGCATGTCAAATACAAATAAACATTACA
TM2128 - pET22 SA2761 XhoI rev	GGTGGTGGTGCTCGAGGATAATGGATAATACTCTG

Supplementary Table S3: LnsA:CPBP family protein complex modeling scoring

LnsA ortholog	*CPBP family protein*	ipTM	pTM
**Heterodimeric complexes [AlphFoldver3 (8)]**			
SAOUHSC_00822	SAOUHSC_02761	0.74	0.77
SAOUHSC_00822	SAOUHSC_01900	0.14	0.54
SAOUHSC_00822	SAOUHSC_02611	0.17	0.48
SAOUHSC_00822	SAOUHSC_02587	0.12	0.53
SAOUHSC_00822	SAOUHSC_00965	0.14	0.49
SAOUHSC_00822	SAOUHSC_02256	0.15	0.52
SAOUHSC_00822	SE_2027	0.71	0.75
SE_0583	SAOUHSC_02761	0.75	0.78
SE_0583	SE_2027	0.75	0.78
SAOUHSC_00822	SCA_1941	0.13	0.53
SAOUHSC_00822	SCA_1942	0.12	0.53
**LnsA (SAOUHSC_00822) with putative signal peptide removed (SP) [AlphFoldver3 (8)]**			
SAOUHSC_00822 (No SP)	SAOUHSC_02761	0.84	0.81
SAOUHSC_00822 (No SP)	SAOUHSC_01900	0.29	0.62
SAOUHSC_00822 (No SP)	SAOUHSC_02611	0.24	0.51
SAOUHSC_00822 (No SP)	SAOUHSC_02587	0.13	0.55
SAOUHSC_00822 (No SP)	SAOUHSC_00965	0.13	0.54
SAOUHSC_00822 (No SP)	SAOUHSC_02256	0.11	0.53
SAOUHSC_00822 (No SP)	SE_2027	0.80	0.77
SE_0583 (No SP)	SAOUHSC_02761	0.81	0.79
SE_0583 (No SP)	SE_2027	0.85	0.83
SAOUHSC_00822 (No SP)	SCA_1941	0.11	0.54
SAOUHSC_00822 (No SP)	SCA_1942	0.16	0.57
**Multimeric LnsA (SAOUHSC_00822, No SP):LnsB (SAOUOHSC_02761) [AlphFoldver3 (8)]**			
2 x SAOUHSC_00822 (No SP)	2 x SAOUHSC_02761	0.32	0.45
3 x SAOUHSC_00822 (No SP)	3 x SAOUHSC_02761	0.25	0.36
4 x SAOUHSC_00822 (No SP)	4 x SAOUHSC_02761	0.23	0.31
5 x SAOUHSC_00822 (No SP)	5 x SAOUHSC_02761	0.22	0.29
6 x SAOUHSC_00822 (No SP)	6 x SAOUHSC_02761	0.22	0.28
**Multimeric LnsA (SAOUHSC_00822, No SP):LnsB (SAOUOHSC_02761) with or without DA-LP ligand [Protenix(9)][a]**			
1 x SAOUHSC_00822 (No SP)	1x SAOUHSC_02761	0.67 (0.58)	0.76 (0.74)
2 x SAOUHSC_00822 (No SP)	2 x SAOUHSC_02761	0.46 (0.53)	0.55 (0.62)
3 x SAOUHSC_00822 (No SP)	3 x SAOUHSC_02761	0.66 (0.34)	0.70 (0.45)
4 x SAOUHSC_00822 (No SP)	4 x SAOUHSC_02761	0.87 (0.64)	0.88 (0.67)
5 x SAOUHSC_00822 (No SP)	5 x SAOUHSC_02761	0.87 (0.60)	0.87 (0.64)
6 x SAOUHSC_00822 (No SP)	6 x SAOUHSC_02761	0.87 (0.69)	0.88 (0.71)

[a] The corresponding complex without ligand is listed in parenthesis for comparison.

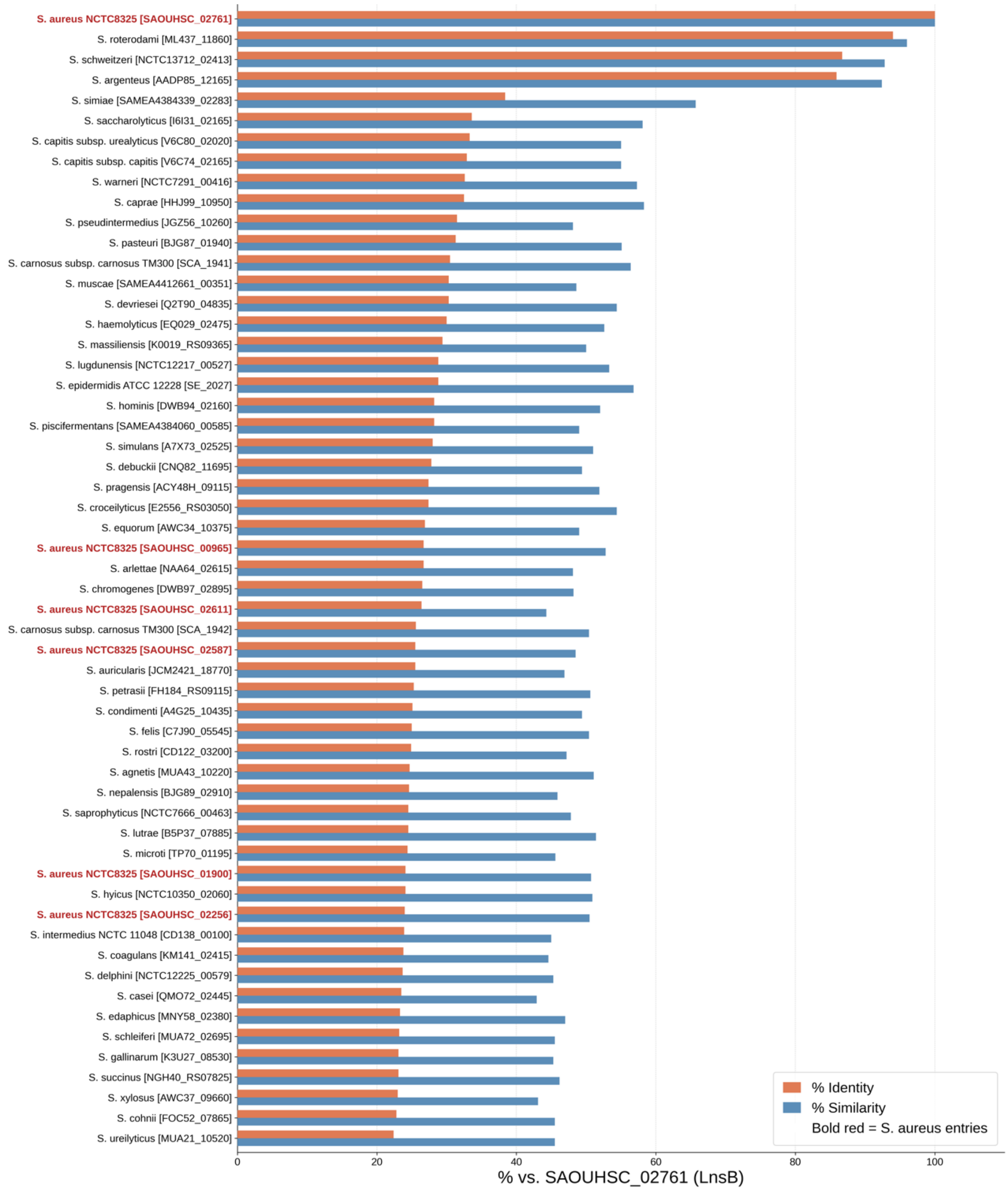

Supplementary Figure 1. Comparison of sequence identity and similarity among CPBP family proteins from staphylococcal species to LnsB (SAOUHSC_02761) from *S. aureus*. A panel of CPBP family proteins, including all CPBP encoding ORFs in *S. aureus* (**bold** text), all syntenic LnsB orthologs from representative strains of *S. epidermidis, S. carnosus*, and *S. casei*, and the top ranking hits from those species lacking syntenic copies were pairwise compared to LnsB (SAOUHSC_02761) from *S. aureus* to obtain %-identity (*red* bars) and %-similarity scores (*blue* bars) using BLOSUM62 (gap open -11, extend -1) pairwise global alignment with Biomni agent interface (10). Gene locus tags are in parenthesis.

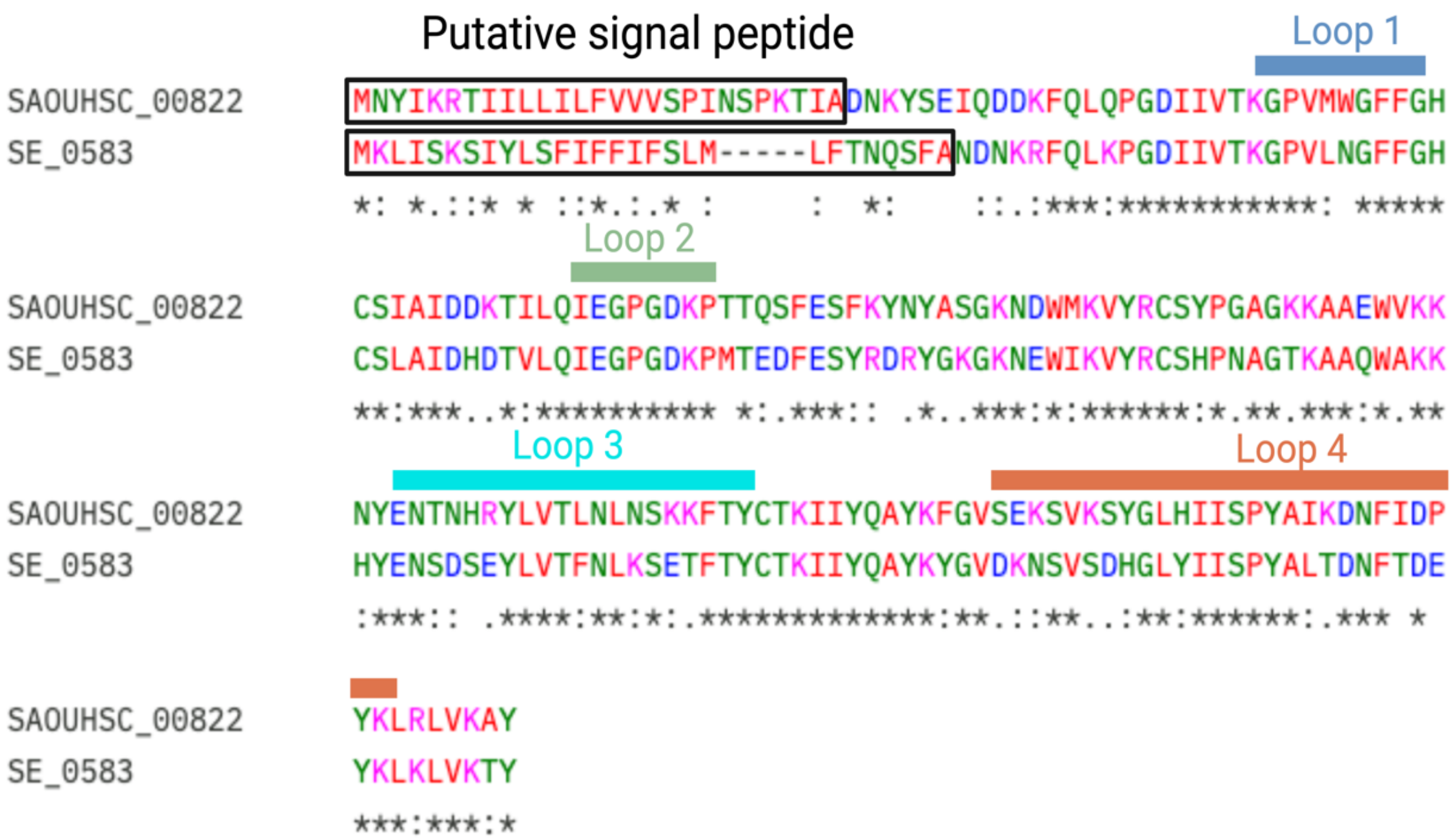

Supplementary Figure 2. Alignment of LnsA orthologs from *S. aureus* (SAOUHSC_00822) and *S. epidermidis* (SE_0583). Protein sequences for LnsA from *S. aureus* and *S. epidermidis* were compared using ClustalOmega (11). The putative signal peptide predicted by SignalP 6.0 (12) is *boxed*, while boundaries for each of the four loops is denoted above the amino acid sequence.

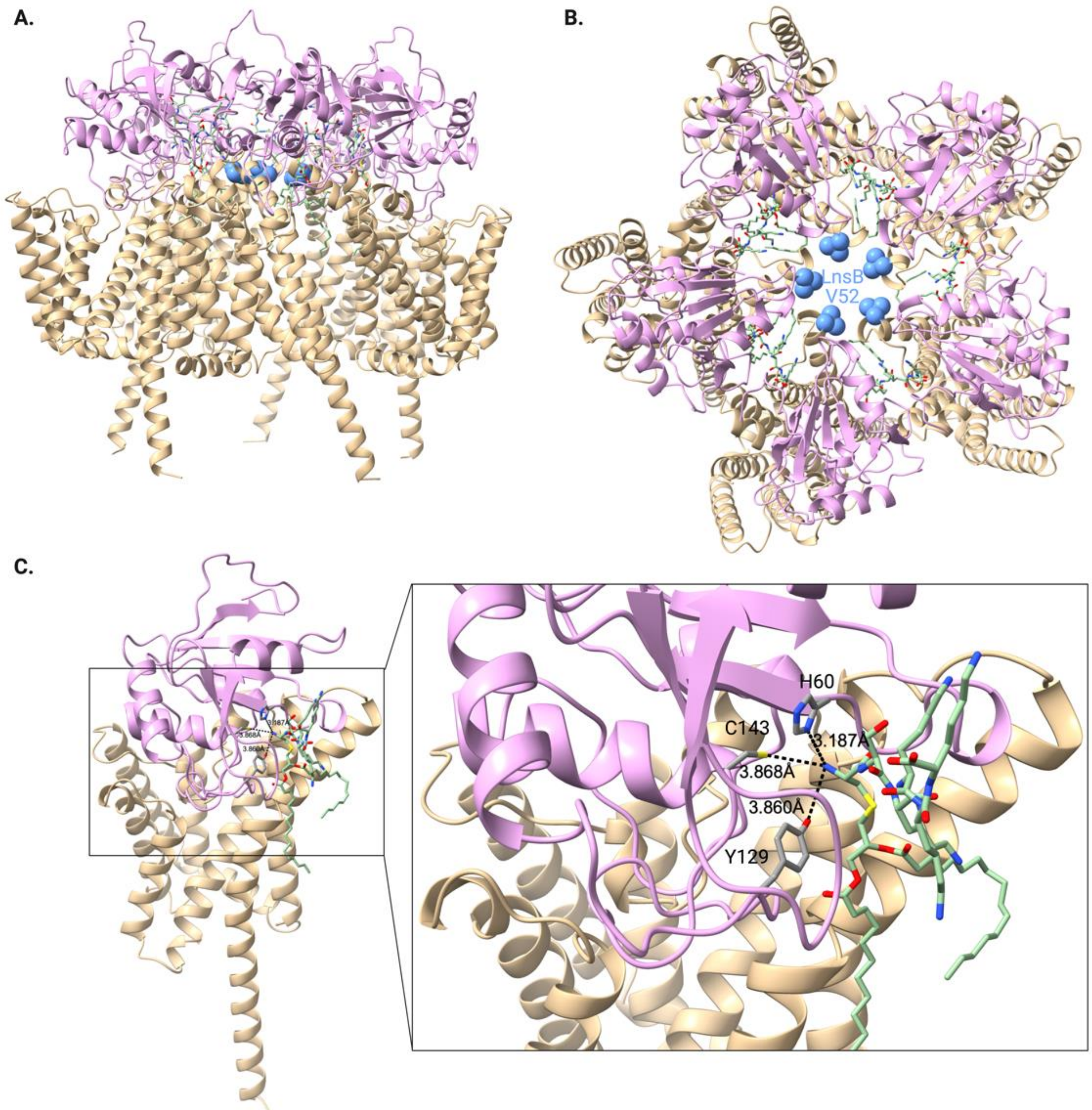

Supplementary Figure 3. Protenix pentameric model of LnsA-LnsB with diacylated lipopeptide. A. Side view of the Protenix model (9) was generated using five copies of each of LnsA (no signal peptide, *plum*) and LnsB (*tan*) with five di-palmitoyl *S*-glyceryl-Cys-Ser-Lys-Lys-Lys-Lys ligand molecules (*green* backbone). Residue Val52 on LnsB (*space filled* representation, *blue*) readily forms homodimeric crosslinks when substituted with cysteine (see **Figure 7**). **B.** *Top-down* view of modeled pentameric complex from outside of membrane surface. **C.** Cutaway of a single LnsA-B heterodimer in complex with ligand bound with a magnified view (*inset*). Distances of the three LnsA residues determined to be essential for catalysis (see **Figure 8**) are labeled relative to the position of the α-amino acyl acceptor on the lipopeptide ligand.

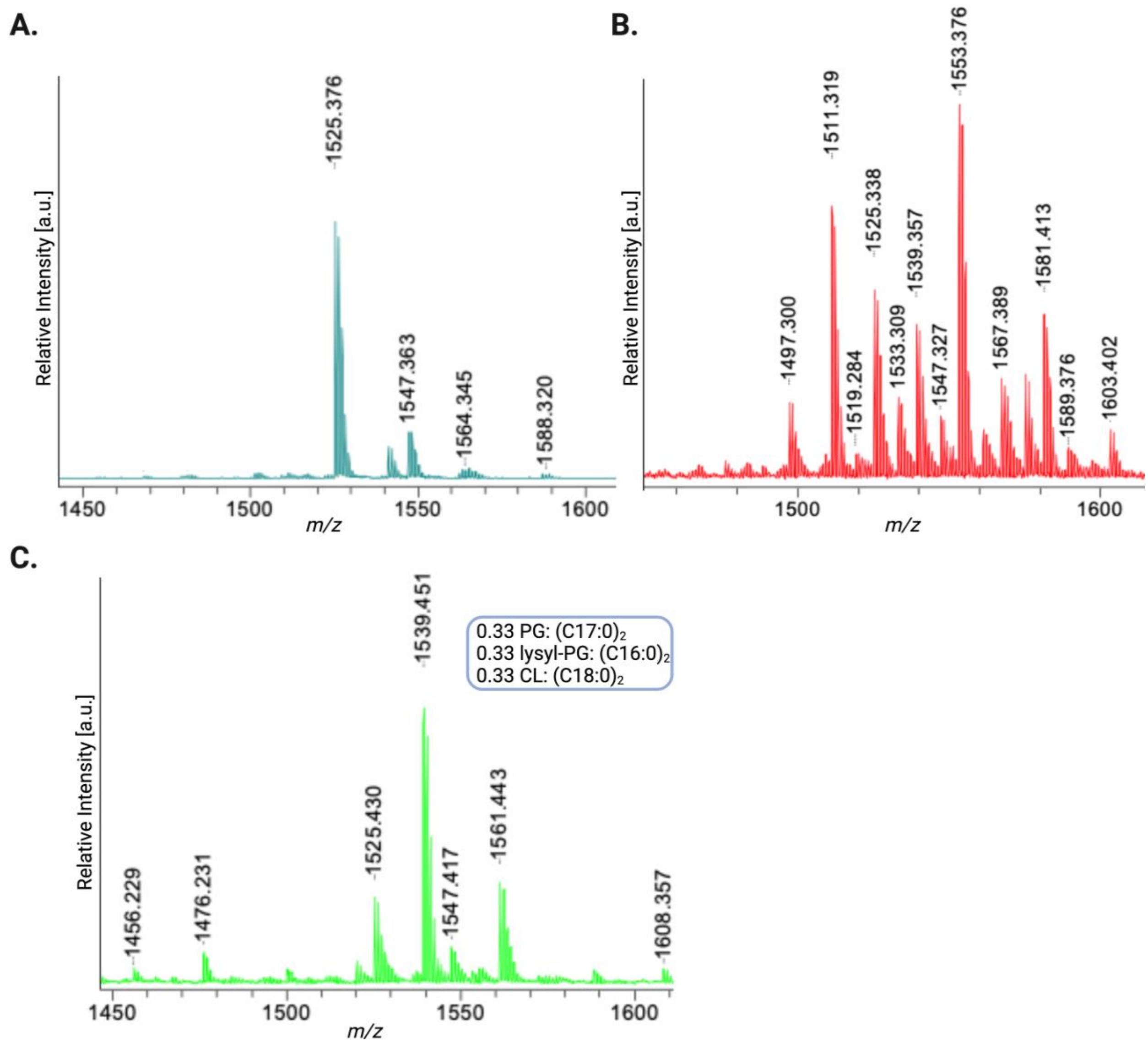

Supplementary Figure 4: Supplemental MALDI-MS Spectra. A. Mass spectra of synthetic TA-LP* (*N*-palmitoyl di-palmitoyl *S*-glyceryl-Cys-Ser-Ser-Gly-Lys-ε-dansyl) standard. **B.** The spectra of TA-LP* product obtained using *S. aureus* total polar phospholipid extracts. **C.** The spectra of TA-LP* arising from a 1:1:1 mole fraction mix of phospholipids (*inset*), with the dominant peak having a mass consistent with C17:0 acyl chain transfer from PG. Peak assignments are listed in **Figure 9D**.

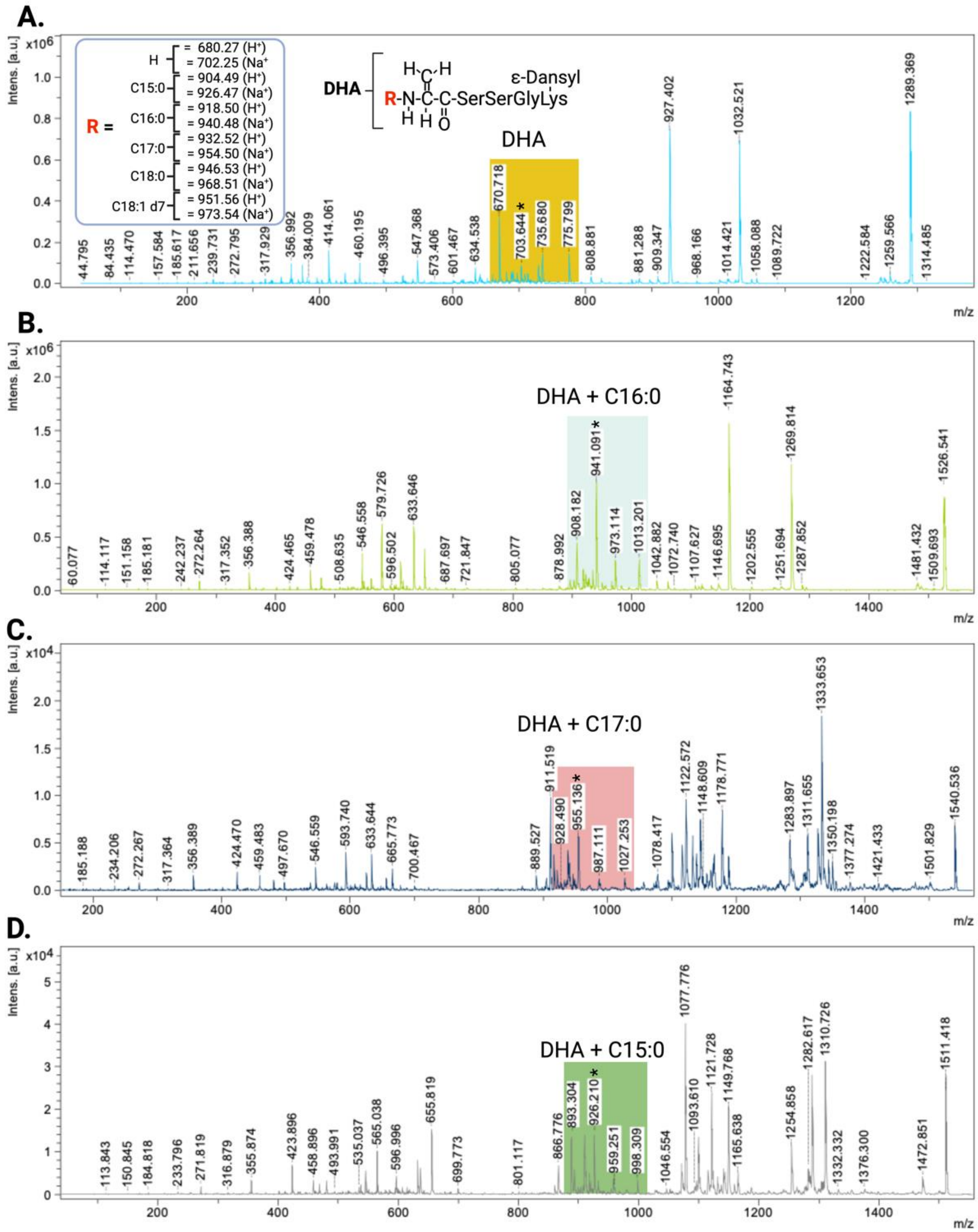

Supplementary Figure 5: MALDI-MS/MS-TOF analysis of the *N*-terminal substitution state of dehydroalanyl (DHA) lipopeptides. MALDI MS/MS spectra were obtained to confirm the identity of the *N*-terminal substitution by analyzing dehydroalanyl (DHA) fragment ions (structure and mass table *inset* of *panel A*) as has been described (13). The DA-LP* standard was consistent with *N*-terminal protonation (parent sodiated mass ion 1286.74 u, panel **A**), the TA-LP* *N*-palmitoyl standard was C16:0 (parent sodiated mass ion 1524.97 u, panel **B**), the product of **Figure 9E** phospholipid mixture was C17:0 (parent sodiated mass ion 1539.98 u, panel **C**), and the product of **Figure 9F** asymmetric PG lipid phospholipid mixture was C15:0 (parent sodiated mass ion 1510.97 u, panel **D**). Masses of DHA derived ions drawn in *panel A* are labeled (*), while other related DHA fragments diagnostic of acylation state are *color* shaded (-33 u, +32 u, +72 u).

REFERENCES

1. Charpentier, E., Anton, A. I., Barry, P., Alfonso, B., Fang, Y., and Novick, R. P. (2004) Novel cassette-based shuttle vector system for Gram-positive bacteria. *Appl. Environ. Microbiol.* 70, 6076–6085
2. Meredith, T. C., Swoboda, J. G., and Walker, S. (2008) Late-stage polyribitol phosphate wall teichoic acid biosynthesis in *Staphylococcus aureus*. *J. Bacteriol.* 190, 3046–3056
3. Gardiner, J. H. t., Komazin, G., Matsuo, M., Cole, K., Gotz, F., and Meredith, T. C. (2020) Lipoprotein *N*-acylation in *Staphylococcus aureus* is catalyzed by a two-component acyl transferase system. *mBio* 11, e01619–20
4. Lee, J. C. (1995) Electrotransformation of staphylococci. In *Electroporation Protocols for Microorganisms* (Nickoloff, J. A., ed) Humana Press, Totowa, NJ, pp. 209–216
5. Kato, F. and Sugai, M. (2011) A simple method of markerless gene deletion in *Staphylococcus aureus*. *J. Microbiol. Methods* 87, 76–81
6. Kho, K. and Meredith, T. C. (2018) Salt-induced stress stimulates a lipoteichoic acid-specific three-component glycosylation system in *Staphylococcus aureus*. *J. Bacteriol.* 200, e00017–18
7. Komazin, G., Wigmore, R. M., Ranade, A. M., Rizk, A. A., Gardiner, J. H. t., and Meredith, T. C. (2025) Lipoprotein *N*-terminal modification in *Bacillus*: a new paradigm for extracellular acetylation and species-dependent Toll-like receptor 2 immunomodulation. *mBio* 16, e0099625
8. Abramson, J., Adler, J., Dunger, J., Evans, R., Green, T., Pritzel, A., Ronneberger, O., Willmore, L., Ballard, A. J., Bambrick, J., et al. (2024) Accurate structure prediction of biomolecular interactions with AlphaFold 3. *Nature* 630, 493–500
9. Team, B. A. A. S., Chen, X., Zhang, Y., Lu, C., Ma, W., Guan, J., Gong, C., Yang, J., Zhang, H., Zhang, K., et al. (2025) Protenix: advancing structure prediction through a comprehensive AlphaFold3 reproduction. *bioRxiv* doi: 10.1101/2025.01.08.631967
10. Huang, K., Zhang, S., Wang, H., Qu, Y., Lu, Y., Roohani, Y., Li, R., Qiu, L., Li, G., Zhang, J., et al. (2025) Biomni: a general-purpose biomedical AI agent. *bioRxiv* doi: 10.1101/2025.05.30.656746
11. Madeira, F., Madhusoodanan, N., Lee, J., Eusebi, A., Niewielska, A., Tivey, A. R. N., Lopez, R., and Butcher, S. (2024) The EMBL-EBI Job Dispatcher sequence analysis tools framework in 2024. *Nucleic Acids Res.* 52, W521–W525
12. Teufel, F., Almagro Armenteros, J. J., Johansen, A. R., Gislason, M. H., Pihl, S. I., Tsirigos, K. D., Winther, O., Brunak, S., von Heijne, G., and Nielsen, H. (2022) SignalP 6.0 predicts all five types of signal peptides using protein language models. *Nat. Biotechnol.* 40, 1023–1025
13. Armbruster, K. M. and Meredith, T. C. (2018) Enrichment of bacterial lipoproteins and preparation of *N*-terminal lipopeptides for structural determination by mass spectrometry. *J. Vis. Exp.* 135, e56842
